# Interrogation of the Structure–Activity Relationship of a Lipophilic Nitroaromatic Prodrug Series Designed for Cancer Gene Therapy Applications

**DOI:** 10.3390/ph15020185

**Published:** 2022-02-01

**Authors:** Amir Ashoorzadeh, Alexandra M. Mowday, Christopher P. Guise, Shevan Silva, Matthew R. Bull, Maria R. Abbattista, Janine N. Copp, Elsie M. Williams, David F. Ackerley, Adam V. Patterson, Jeff B. Smaill

**Affiliations:** 1 Auckland Cancer Society Research Centre, School of Medical Sciences, The University of Auckland, Auckland 1142, New Zealand; a.ashoorzadeh@auckland.ac.nz (A.A.); a.mowday@auckland.ac.nz (A.M.M.); chrispguise@hotmail.com (C.P.G.); shevansilva116@gmail.com (S.S.); m.bull@auckland.ac.nz (M.R.B.); m.abbattista60@gmail.com (M.R.A.); a.patterson@auckland.ac.nz (A.V.P.); 2 Maurice Wilkins Centre for Molecular Biodiscovery, The University of Auckland, Auckland 1142, New Zealand; janine.copp@msl.ubc.ca (J.N.C.); elsie.williams@vuw.ac.nz (E.M.W.); david.ackerley@vuw.ac.nz (D.F.A.); 3 School of Biological Sciences, Victoria University of Wellington, Wellington 6140, New Zealand; 4 Michael Smith Laboratories, University of British Columbia, Vancouver, BC V6T 1Z4, Canada

**Keywords:** nitrobenzamide, mustard, prodrug, SAR, bystander effect, cancer gene therapy, nitroreductase, AKR1C3

## Abstract

PR-104A is a dual hypoxia/nitroreductase gene therapy prodrug by virtue of its ability to undergo either one- or two-electron reduction to its cytotoxic species. It has been evaluated extensively in pre-clinical GDEPT studies, yet off-target human aldo-keto reductase AKR1C3-mediated activation has limited its use. Re-evaluation of this chemical scaffold has previously identified SN29176 as an improved hypoxia-activated prodrug analogue of PR-104A that is free from AKR1C3 activation. However, optimization of the bystander effect of SN29176 is required for use in a GDEPT setting to compensate for the non-uniform distribution of therapeutic gene transfer that is often observed with current gene therapy vectors. A lipophilic series of eight analogues were synthesized from commercially available 3,4-difluorobenzaldehyde. Calculated octanol-water partition coefficients (LogD_7.4_) spanned > 2 orders of magnitude. 2D anti-proliferative and 3D multicellular layer assays were performed using isogenic HCT116 cells expressing *E. coli* NfsA nitroreductase (NfsA_Ec) or AKR1C3 to determine enzyme activity and measure bystander effect. A variation in potency for NfsA_Ec was observed, while all prodrugs appeared AKR1C3-resistant by 2D assay. However, 3D assays indicated that increasing prodrug lipophilicity correlated with increased AKR1C3 activation and NfsA_Ec activity, suggesting that metabolite loss from the cell of origin into media during 2D monolayer assays could mask cytotoxicity. Three prodrugs were identified as bono fide AKR1C3-negative candidates whilst maintaining activity with NfsA_Ec. These were converted to their phosphate ester pre-prodrugs before being taken forward into in vivo therapeutic efficacy studies. Ultimately, 2-(5-(bis(2-bromoethyl)amino)-4-(ethylsulfonyl)-*N*-methyl-2-nitrobenzamido)ethyl dihydrogen phosphate possessed a significant 156% improvement in median survival in mixed NfsA_Ec/WT tumors compared to untreated controls (*p* = 0.005), whilst still maintaining hypoxia selectivity comparable to PR-104A.

## 1. Introduction

Cancer gene therapy involves the transfer of foreign genetic material into target cells for therapeutic benefit. In the case of gene-directed enzyme prodrug therapy (GDEPT), a transcriptional unit encoding an exogenous enzyme is delivered to cells, expression of which is able to sensitize transfected tumor cells to an otherwise inert prodrug [1,2]. Localized catalytic activation of prodrug in this manner creates an improved therapeutic index and, in principle, can generate active drug concentrations at levels that are unachievable with non-targeted, traditional chemotherapy agents. Depending on the tissue penetration capacity of the active metabolite(s), cytotoxic products can diffuse into and kill metabolically naïve neighboring cells, a phenomenon termed the ‘bystander effect’ [3,4]. Heterogeneous vector distribution and/or the constrained vector geometry observed with current gene delivery vectors necessitates the optimization of prodrug bystander activity in order to compensate for the non-uniform distribution of gene transfer, thereby facilitating tumor control when only a subset of cells expresses the prodrug activating enzyme [5,6].

The most clinically advanced enzyme/prodrug combination for GDEPT to date is herpes simplex virus thymidine kinase (HSV-tk) in combination with ganciclovir, which reached phase III clinical trial [7]. Whilst ganciclovir can diffuse freely into cells, the cytotoxic metabolite of ganciclovir (ganciclovir triphosphate) is charged at physiological pH, and thus is membrane-impermeable [8]. Consequently, bystander effect is dependent on gap-junctional intracellular communication for metabolite passage between cells, which can be downregulated in many tumor types [9]. However, there are alternative enzyme/prodrug combinations that have demonstrated significant bystander activity in vitro and in vivo, including the nitroaromatic mustard family of prodrugs in combination with bacterial nitroreductase (NTR) enzymes [10,11]. A major advantage to this class of compound is the stability of the cytotoxic products following reduction; the active metabolites have an appreciable half-life and are able to diffuse out of the cell of origin, resulting in bystander kill of neighboring cells.

**Figure 1:**
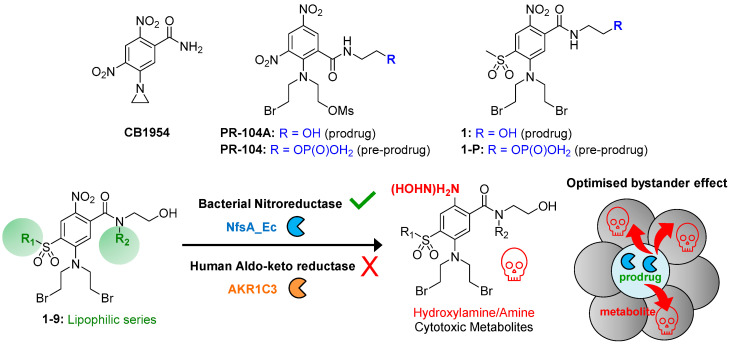
Structures of known prodrug examples and rationale for the present work.

The prototypical nitroaromatic enzyme/prodrug combination for GDEPT is the nitroreductase from *Escherichia coli*, NfsB (NfsB_Ec), in combination with the prodrug CB1954 (Figure 1) [12]. NfsB_Ec has also been partnered with the dual hypoxia/NTR prodrug PR-104 in preclinical models with considerable success [13]. PR-104 is a 3,5-dinitrobenzamide mustard phosphate ester that undergoes systemic hydrolysis to the alcohol prodrug PR-104A [14]. Cellular one-electron oxidoreductases initiate conversion of the nitro group of PR-104A to the hydroxylamine and amine (PR-104H and PR104M, respectively) under low-oxygen conditions, activating a latent nitrogen mustard moiety that can sterilize hypoxic cells. Two-electron reduction by the E. coli NfsB nitroreductase (NfsB_Ec) can reduce PR-104A directly to cytotoxic metabolites in an oxygen-independent manner. Recently, alternative bacterial NTRs have been identified as having activity with PR-104A and CB1954 [15,16,17]. Both of these prodrugs are not without limitations, however. CB1954 is hindered by poor aqueous solubility [18] and dose-limiting hepatotoxicity in humans [19]. For PR-104A, off-mechanism activation by human aldo-keto reductase 1C3 (AKR1C3) is considered to contribute to the dose-limiting myelosuppression observed in humans, restricting exposure to approximately 10–29% of that achievable in mice [20,21,22].

Recently, we reported a novel analogue of PR-104A, SN29176 (1, Figure 1), that eliminates off-mechanism activation by human AKR1C3 whilst maintaining hypoxia selectivity in vitro [22]. This was confirmed in vivo when 1 was administered as its water-soluble phosphate ester pre-prodrug SN35141 (1-P, Figure 1). In the present study, we describe the experimental procedures for an improved synthetic route to 1 and characterize the potential of this compound as a bacterial nitroreductase prodrug for GDEPT applications. In addition, we synthesized a series of novel, lipophilic analogues of 1, seeking to identify a lead candidate for GDEPT, with an optimized bystander effect, that remains free from AKR1C3 activation. We describe nine compounds, with the lipophilicity of each compound able to be modified in two positions on the chemical scaffold. We interrogated the structure–activity relationship of these compounds in two (2D) and three dimensions (3D) using cell lines expressing the major NTR from *E. coli*, NfsA, or human AKR1C3, before identifying three preferred structures. We then compared the in vivo efficacy of the three leads (as their phosphate ester pre-prodrugs) in a xenograft model containing a minority percentage of NfsA-expressing cells and confirmed that the final lead compound (6/6-P) retains hypoxia selectivity in vitro and in vivo.

## 2. Results

### 2.1. Drug Design Rationale

In a GDEPT setting, heterogeneous gene therapy vector distribution and/or constrained vector geometry necessitates the optimization of prodrug bystander activity. Increased prodrug lipophilicity (LogD_7.4_, typically measured as the octanol:water partition coefficient) has been correlated with improved bystander efficiency [4]. Having previously identified compound **1** as a hypoxia-activated prodrug that was free from off-mechanism activation by human AKR1C3 [22], we reasoned this was an ideal scaffold to utilize for the generation of a series of lipophilic analogues. This was achieved through introduction of a range of alkyl substituents at the sulfone (R_1_) and carboxamide nitrogen (R_2_) positions of the scaffold (Table 1).

### 2.2. Chemical Synthesis

**Scheme 1:**
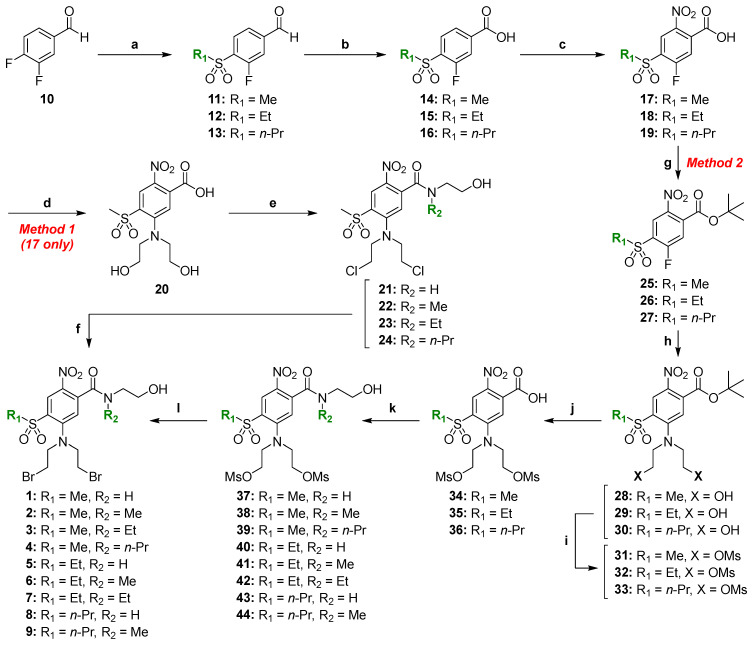
Synthesis of compounds **1**–**9** via two methods. Reagents and conditions: (**a**) sodium alkyl sulfinate, DMSO, 75 °C, 3 h; (**b**) NaH_2_PO_4_.4H_2_O and c. HCl (buffer), H_2_O_2_, NaClO_2_, water, CH_3_CN, 0 °C to R.T., 5 h; (**c**) Fum. HNO_3_, c. H_2_SO_4_, R.T. to 45 °C, 4 h; (**d**) diethanolamine, DMSO, R.T., 2 h; (**e**) (i) SOCl_2_, cat. DMF, reflux, 4 h, (ii) ethanolamine or alkyl ethanolamine, CH_2_Cl_2_, THF, 0 °C to R.T., 20 min; (**f**) LiBr, 3-methyl-2-butanone, reflux, 12 h (3×); (**g**) *tert*-butyl acetate, HClO_4_ (70%), CH_3_CN, R.T., 48 h; (**h**) diethanolamine, DMSO, R.T., 2 h; (**i**) MsCl, Et_3_N, CH_2_Cl_2_, 0 °C to R.T., 20 min; (**j**) TFA, CH_2_Cl_2_, R.T., 2 h; (**k**) (i) oxalyl chloride, cat. DMF, MgO, CH_2_Cl_2_, CH_3_CN, 0 °C to R.T., 4 h; (ii) ethanolamine or alkyl ethanolamine, 0 °C to R.T., 20 min; (**l**) LiBr, acetone, R.T., 12 h.

Synthesis of intermediate 17 has been previously described by our group during the preparation of the hypoxia-activated prodrug CP-506 [23] and is briefly included here for completeness. Key intermediates 17–19 were prepared by nucleophilic displacement of the 4-fluoro substituent of commercially available 3,4-difluorobenzaldehyde (10) with the respective sodium alkyl sulfinates to give the alkyl sulfones 11–13, followed by oxidation to afford the carboxylic acids 14–16 and subsequent nitration mediated by fuming nitric acid (Scheme 1).

Two alternate methods were developed for the synthesis of the final test compounds. We have previously reported the synthesis of 1 in three steps from intermediate 17 via Method 1 (i.e., 17 to 20 to 21 to 1) [22]. This route was also utilized to prepare compounds **2**–**4** in the present paper. Briefly, the alcohol and carboxylic acid functionalities of diol 20 [22] were concurrently chlorinated with thionyl chloride before the resultant acid chloride intermediate was coupled with various amine side chains to give the carboxamides 22–24. Halogen exchange mediated by lithium bromide in methyl isopropyl ketone at reflux afforded the respective dibromo mustards 2–4 (Scheme 1). Analytical HPLC and ^1^H NMR analyses of compounds **2**–**4** demonstrated that they exist as a mixture of atropisomers [24] at ambient temperature. A well-described phenomena for tertiary aromatic amides bearing an ortho substituent wherein the aromatic ring and the amide plane are near perpendicular [25,26]. Hindered rotation about the amide bond results in two conformations that can interconvert slowly on an NMR timescale at ambient temperature and are hence detectable by NMR and characterized by chemical exchange effects in NMR experiments [27,28,29].

**Scheme 2:**
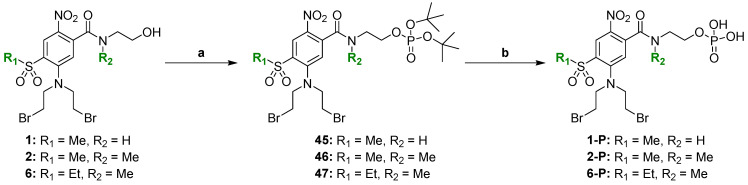
Synthesis of the phosphate ester pre-prodrugs 1-P, 2-P, and 6-P. Reagents and conditions: (**a**) (i) di-*tert*-butyl-*N*,*N*-diisopropylphosphoramidite, 1*H*-tetrazole (3% in CH_3_CN), DMF, 5 °C to R.T., 4 h; (ii) *m*-CPBA (70%), CH_2_Cl_2_, 0 °C to R.T., 1 h; (**b**) TFA, CH_2_Cl_2_, 5 °C to R.T., 1 h.

Method 1 as described in Scheme 1 provided a relatively short synthetic route to access a range of symmetrical mustard analogues. Nevertheless, the method has potential disadvantages, including a low overall yield and the use of rigorous reaction conditions in the final Finkelstein halogen exchange step [30,31]. In order to overcome these limitations, we designed an alternative route (Method 2), employing a carboxylic acid protecting group strategy [32]. Compounds **1**, **2**, and **4** were resynthesized through employing Method 2 (Scheme 1), while compounds **5**–**9** were also prepared via this method. The carboxylic acid group of key intermediates 17–19 was protected as the *tert*-butyl ester to give 25–27. Nucleophilic displacement of the respective activated fluorine by diethanolamine gave the diols 28–30. Reaction with mesyl chloride gave mesylates 31–33 before trifluoroacetic acid-mediated deprotection gave the carboxylic acids 34–36. Conversion of carboxylic acids 34–36 to their respective acid chloride intermediates was performed at ambient temperature using oxalyl chloride in the presence of MgO to avoid undesired displacement of the mesylate groups by chloride ions. The freshly prepared acid chlorides were then coupled with various amines in situ to give the amides 37–44. Mesylate displacement of 37–44 employing lithium bromide in acetone at room temperature then afforded the respective dibromo mustards 1, 2, and 4–9 (Scheme 1). Synthesis of the phosphate pre-prodrug 1-P via intermediate 45 has been previously described [22] and is included here for completeness. Phosphate pre-prodrugs 2-P and 6-P were prepared by the same method. Reaction of their respective alcohols (2 and 6) with di-*tert*-butyl-*N*,*N*-diisopropylphosphoramidite and subsequent oxidation gave the di-*tert*-butyl phosphate esters 46 and 47, respectively. TFA-mediated deprotection then gave the pre-prodrugs 2-P and 6-P, respectively (Scheme 2).

### 2.3. Anti-Proliferative Activity In Vitro

**Figure 2:**
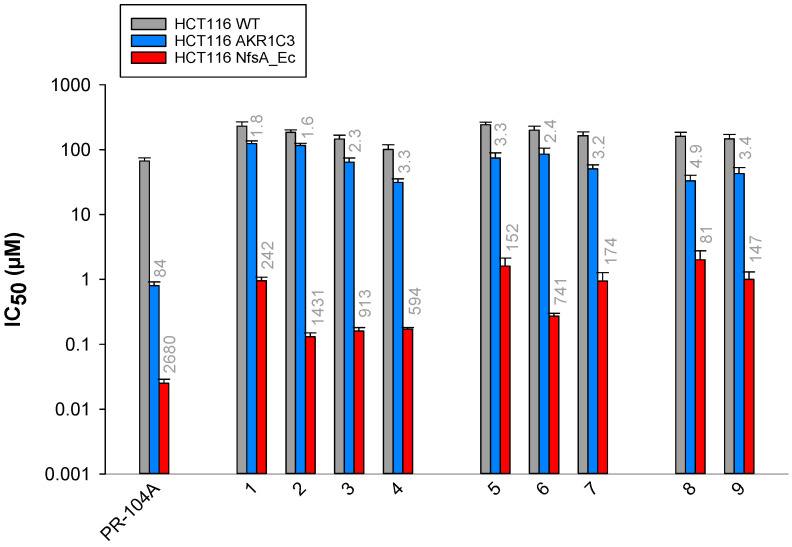
In vitro anti-proliferative activity of the novel lipophilic analogue series in HCT116 cells expressing AKR1C3 and *E. coli* NfsA (NfsA_Ec). IC_50_ values were determined as the concentration of drug required to inhibit cell growth by 50% of untreated controls following four hour drug exposure, with washing and regrowth for five days. Respective IC_50_ ratios (WT:AKR1C3 or WT:NfsA_Ec) are indicated above the bars for each compound.

Next, we evaluated the compounds for activity with the major nitroreductase from *E. coli*, NfsA (NfsA_Ec), or the human aldo-keto reductase AKR1C3, using a low cell density anti-proliferative (IC_50_) assay (Figure 2). The sensitivity of HCT116 wild-type (WT) cells and HCT116 cells over-expressing either NfsA_Ec or AKR1C3 was examined under aerobic conditions. A range of potency for NfsA_Ec was observed, with IC_50_ values ranging from 0.13 to 2.0 µM, and corresponding WT:NfsA_Ec IC_50_ ratios ranging from 81- to 1431-fold. However, none of the novel scaffolds demonstrated an improvement in NfsA_Ec IC_50_ relative to PR-104A (0.03 µM IC_50_ value in NfsA_Ec cells, WT:NfsA_Ec IC_50_ ratio of 2680-fold). Interestingly, anti-proliferative activity with NfsA_Ec appeared to decrease when there was a hydrogen in the R2 position (1, 5, and 8). A moderate negative linear relationship (R = −0.4, R^2^ = 0.14) was observed between the WT:NfsA_Ec IC_50_ ratios and LogD_7.4_, suggesting that the more lipophilic compounds in the analogue series have decreased NfsA_Ec activity (Figure S1). All compounds appeared to be minimal substrates for AKR1C3, with WT:AKR1C3 IC_50_ ratios of less than five across the series (in comparison to a ratio of 84-fold for PR-104A).

### 2.4. Interrogation of the Lipophilic SAR in Three Dimensions

**Figure 3:**
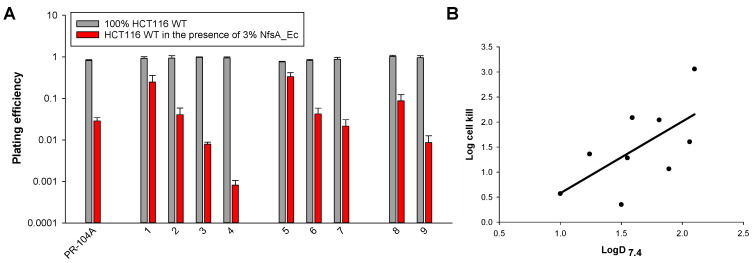
Lipophilic structure–activity relationship for NfsA_Ec in three dimensions. (**A**) Plating efficiency in 100% HCT116 WT and 97% HCT116 WT/3% HCT116 NfsA_Ec MCLs exposed to 10 µM prodrug for five hours before dissociation and plating to determine clonogenic survival. (**B**) Correlation between Log cell kill and LogD_7.4_. R = 0.63, R^2^ = 0.40. Log cell kill = Log CS (control)–Log CS (treated), where CS is the number of clonogenic survivors per MCL.

We then examined our compounds in a high cell density, 3D multi-cellular layer (MCL) assay in order to determine tissue penetration capacity of the active metabolite(s) and the potential for an efficient bystander effect [4]. Mixed MCLs consisting of a small percentage HCT116 NfsA_Ec cells (3%) in the presence of HCT116 WT cells (97%) were used as a robust screen for efficient bystander cell kill. Here, active cytotoxic metabolites can diffuse out of the minority NfsA_Ec ‘prodrug-activating’ cell population into the WT ‘target’ population, causing death of cells that would not normally be sensitive to that concentration of prodrug. The 97% HCT116 WT/3% HCT116 NfsA_Ec MCLs were sensitive to all prodrugs at 10 µM, demonstrating log cell kill values (LCK) of between 0.35 and 3.06 (Figure 3). Overall, increasing LogD_7.4_ was shown to correlate with increasing LCK (R = 0.63, R^2^ = 0.4), indicating that lipophilicity plays a role in the overall bystander efficacy of these compounds at tissue-like cell densities.

**Figure 4:**
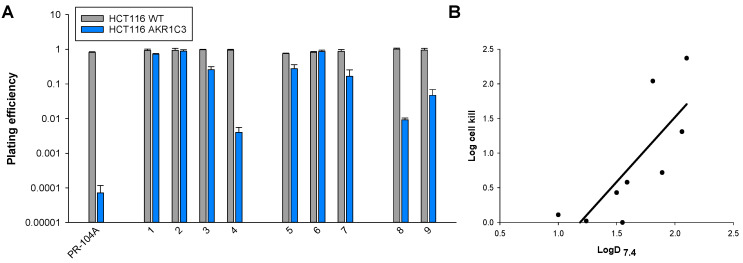
Lipophilic structure–activity relationship for AKR1C3 in three dimensions. (**A**) Plating efficiency in 100% HCT116 WT and 100% HCT116 AKR1C3 MCLs exposed to 10 µM prodrug for five hours before dissociation and plating to determine clonogenic survival. (**B**) Correlation between Log cell kill and LogD_7.4_. R = 0.78, R^2^ = 0.61. Log cell kill = Log CS (control)–Log CS (treated), where CS is the number of clonogenic survivors per MCL.

Screening for AKR1C3 activity at high cell density indicated that only three compounds appeared to be non-substrates at 10 μM prodrug, demonstrating LCK values of ≤ 0.11 (1, 2, and 6; Figure 4). Similar to the 3D NfsA_Ec screen, increased lipophilicity also tracked with increased LCK in 100% AKR1C3-expressing MCLs (R = 0.78, R^2^ = 0.61). Overall, three lead prodrugs from this analogue series successfully fulfilled the criteria of being resistant to AKR1C3 activation whilst demonstrating observable NfsA_Ec-mediated bystander cell killing in vitro (1, 2, and 6). These three lead compounds were taken forward into in vivo studies.

### 2.5. Activity of the Lead Compounds against NfsA_Ec-Expressing Tumors

NfsA_Ec potency and bystander effect from the diffusion of activated metabolites is only one determinant of the therapeutic utility of a GDEPT prodrug. Overall, anti-tumor activity will also depend on the efficiency of metabolic activation at well-tolerated prodrug doses, determined mainly by the relationship between host toxicity and plasma/tumor pharmacokinetics. Like PR-104A, the three lead prodrugs (1, 2, and 6) contain an alcohol side chain that requires addition of a phosphate ester to increase water solubility for in vivo applications [14]. We previously determined the maximum tolerated dose (MTD) of the phosphate pre-prodrug 1-P in nude mice to be 1780 µmol/kg (i.p.) [22]. Employing identical methods, we determined the MTD of compounds 2-P and 6-P to be 1000 µmol/kg and 1330 µmol/kg, respectively (data summary in Table S1). In preparation for efficacy testing against H1299 xenografts, we next determined the MTD of each compound (1-P, 2-P, and 6-P) in H1299 tumor-bearing mice to be 1330 µmol/kg, 750 µmol/kg, and 1330 µmol/kg, respectively (Table S1). In each case 1-P, 2-P, and 6-P administration (i.p.) was well tolerated, with no evidence of morbidity or mortality, accompanied by a minimal body weight loss (BWL) nadir of −2.2%, −4.0%, and −5.3%, respectively (MTD defined as <−15% average BWL).

H1299 cells expressing NfsA_Ec were confirmed to demonstrate activity with the three lead prodrug compounds (Table S2). WT:NfsA_Ec IC_50_ ratios of 141-, 1029-, and 472-fold were observed in this cell line for 1, 2, and 6, respectively, in comparison to 2848-fold for PR-104A. These ratios were broadly similar to those observed in HCT116 cells (Figure 2). A mixed H1299 xenograft model containing a minority percentage (28 ± 3.3%) of NfsA_Ec-expressing cells was used in order to rank the efficacy of the prodrugs in vivo. A model such as this necessitates the presence of a robust bystander effect to achieve a tumor growth delay. Tumor-bearing mice were administered a single intraperitoneal dose of prodrugs 1-P, 2-P, and 6-P at the H1299 tumor-bearing MTD. PR-104 was administered at 388 µmol/kg, the human equivalent dose (HED) in mice that provides a plasma AUC_free_ of PR-104A (38 µM-h) that equates to the human Q3W MTD of 1100 mg/m^2^ [22].

**Figure 5:**
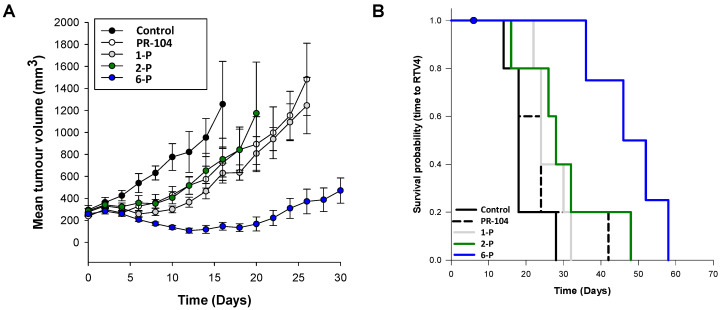
In vivo efficacy of the three lead pre-prodrug compounds in a mixed H1299 xenograft containing 28% NfsA_Ec cells and 72% WT cells. (**A**) Tumor growth and (**B**) survival analysis of mice bearing 250 mm^3^ H1299 tumors treated with a single intraperitoneal dose of prodrug on day 0. N = 5 animals per group. Control = PBS, PR-104 = 388 µmol/kg; 1-P = 1330 μmol/kg; 2-P = 750 μmol/kg; and 6-P = 1330 μmol/kg. Survival endpoint was 4× start volume (RTV4).

Control tumors received vehicle only and grew progressively, reaching RTV4 (a fourfold increase in volume relative to pre-treatment volume) in a median time of 18 days (Figure 5). PR-104-treated tumors produced a modest growth delay of 33% compared to untreated controls, but this was not significant (*p* = 0.40, Log-rank test). Pre-prodrugs 1-P and 2-P did not produce an improvement in survival time compared to PR-104 treated tumors. Pre-prodrug 6-P was the only compound to demonstrate an improvement in survival time when compared to both untreated and PR-104 treated groups (tumor growth delays of 156% (*p* = 0.005) and 92% (*p* = 0.019), respectively, Log-rank test; Figure 5B). All tumors grew to endpoint within the experiment.

We next sought to characterize the NfsA_Ec-mediated conversion of lead prodrug 6 in steady-state kinetics assays using purified His_6_-tagged NfsA_Ec. Compound **6** demonstrated a *k_cat_/K_M_* of 27 ± 5 mM^−1^ s^−1^ (Table S3), confirming it as a less efficient NfsA_Ec substrate than PR-104A [15]. We therefore conducted a preliminary assessment of whether alternative bacterial NTR enzymes might hold promise for further GDEPT improvements. Screening for activation of the SOS (DNA damage repair) response in a library of *E. coli* reporter cells expressing 47 different NTR candidates [16] revealed that compound **6** is broadly activated by members of both the NfsA and NfsB nitroreductase families, and that several members of each family are more effective than NfsA_Ec (Figure S2). This suggests there are prospects for further improvements to a GDEPT system employing prodrug 6 by varying the nitroreductase partner.

### 2.6. Hypoxia Selectivity of 6 and 6-P

**Figure 6:**
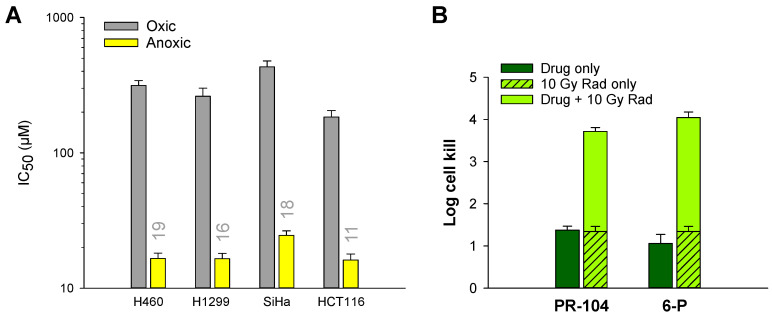
Evaluation of the hypoxia selectivity of lead prodrug 6 in vitro and in vivo (administered as 6-P). (**A**) In vitro anti-proliferative activity of 6 in various cancer cell lines under aerobic and anoxic conditions. IC_50_ values were determined as the concentration of drug required to inhibit cell growth by 50% of untreated controls following 4 h drug exposure and five days regrowth (mean ±SEM of three or more independent determinations). (**B**) Clonogenic cell kill of SiHa xenografts 18 h after treatment with hypoxia activated prodrugs PR-104 (380 µmol/kg, i.p.) or 6-P (1330 µmol/kg, i.p.) with or without 10 Gy radiation.

Having identified 6 (and its corresponding phosphate 6-P) as the lead GDEPT prodrug, we next sought to determine the extent of its activation in hypoxic cells. The sensitivity of four cancer cell lines to prodrug 6 was determined under aerobic or anoxic conditions using an anti-proliferative IC_50_ assay. Hypoxia cytotoxicity ratios (HCRs; aerobic IC_50_ value divided by anoxic IC_50_ value) ranged from 11- to 19-fold (Figure 6A). We also evaluated the hypoxic activity of 6-P versus PR-104 in vivo using a SiHa xenograft model (Figure 6B). As a single agent, PR-104 displayed similar efficacy to 6-P (1.38 vs. 1.06 log cell kill; *p* = 0.31, Student’s *t*-test), despite the fact that SiHa cells express AKR1C3 (thus enabling oxygen independent activation of PR-104 throughout the tumor). Single agent activity of 6-P is consistent with prodrug activation in the hypoxic compartment of the tumor followed by metabolite bystander effect. Comparable levels of hypoxic cell kill were also observed for PR-104 and 6-P in combination with radiotherapy (3.72 vs. 4.05 log cell kill; *p* = 0.12, Student’s *t*-test), with one of the four tumors treated with 6-P showing off-scale activity in this model (less than 1 clonogen per 300K cells).

## 3. Discussion

The finding that PR-104A displays off-mechanism activation by human AKR1C3 has limited its development as a hypoxia/NTR activated prodrug for GDEPT, despite several attractive features. This stimulated the discovery of SN29176 (1) as a potential alternative hypoxia-activated prodrug free of AKR1C3 activation [22]. In the present work, we investigated the potential of 1 for use as a prodrug in a GDEPT setting employing NfsA_Ec as the bacterial nitroreductase of interest, on the basis of its potential to be imaged in the tumor microenvironment by positron emission tomography [33]. Herein, we detail a new route for the chemical synthesis of 1 alongside a series of novel lipophilic mustard analogues 2–9, generated to be free from AKR1C3 activity, and yet still optimized for prodrug bystander activity, an important component of GDEPT technologies. The synthesis employed two methods that pivot at the common nitrobenzoic acid intermediates 17–19. Method 1 includes a low yielding trichlorination of intermediate 20 followed by an acid chloride coupling and high temperature halogen exchange that required repeated submissions to the reaction conditions to achieve completion. Method 2 sought to first protect intermediates 17–19 before introducing a bis-mesylate mustard. Deprotection and amide coupling was then followed by room temperature mesylate displacement by bromide ions. Despite being three steps longer, Method 2 was considered the preferred route to the target compounds. Compounds **1**, **2**, and **4** were prepared from 17 via both methods, and in each case, Method 2 provided a higher overall yield (40%, 29%, and 33%, respectively) than Method 1 (38%, 19%, and 18%, respectively).

All compounds demonstrated anti-proliferative activity with NfsA_Ec in monolayer cultures, and it appeared that as lipophilicity increased across the series, the WT:NfsA_Ec IC_50_ ratios (NfsA_Ec-mediated cytotoxicity) decreased (Figure S1). This observation conflicted with previously generated data reported by Atwell et al., where cytotoxic potency achieved in low cell density cultures correlated positively with LogD_7.4_ [34]. However, this disparity may reflect benzenecarboxamide regioisomer differences (only 2 of 32 compounds in the Atwell study were 1,2,4,5-substituted as in the present series). It may also have been due to a lower range of LogD_7.4_ values used (average LogD_7.4_ = 0.8; median = 1.0, range = −1.5–2.4) in comparison to this analogue series (average LogD_7.4_ = 1.6; median = 1.6, range = 1–2.1). Notably, the loss of potency was most apparent for compounds with an n-propyl extension in the R1 position (8 and 9).

It is now widely accepted that three-dimensional (3D) cell culture systems that mimic key aspects of tissue structure are more representative of the in vivo environment than simple two-dimensional (2D) monolayer cell culture assays. When compounds are tested for an efficient bystander effect, it is possible that the large extracellular dilution effects that are present in a monolayer setting are contributing to an under-estimation of potency for more lipophilic compounds due to cytotoxic metabolite ‘wash out’ (loss) into the excess of cell culture media. This phenomenon arises as a consequence of the ≥10^5^-fold difference between total cell volume (e.g., 10^3^ cells ≈ 1 nL) and total media volume (media volume ≈ 200,000 nL) in an individual test well of a typical 96-well plate. To test this hypothesis, we examined our compounds in a high cell density, 3D multi-cellular layer (MCL) assay. In this setting, any lipophilic cytotoxic metabolites generated will diffuse into a neighboring cell rather than being lost to essentially infinite dilution in the large volume of extracellular media. In direct contrast to the 2D anti-proliferative assay, there was a trend towards increased NfsA_Ec-mediated cytotoxicity with increasing LogD_7.4_, particularly when the lipophilicity remained constant at the R1 position but was increased incrementally at the R2 position. This was in accordance with other SAR studies on a related dinitrobenzamide mustard class of prodrugs [4,34]. For this particular analogue series, compound **4** had the best bystander potential (LCK = 3.06, LogD_7.4_ = 2.1). However, a compound with an equivalent LogD_7.4_ to 4, compound **9**, did not perform as well in this assay (LCK = 1.6), suggesting that there is an additional SAR involved, most likely substrate specificity for the NfsA_Ec active site (IC_50_ values for these substrates were 0.17 and 1.0 µM, respectively). The absence of a relationship between NfsA_Ec-mediated cytotoxicity in two and three dimensions indicates that IC_50_ determinations of potency in low cell density formats alone are not sufficient to rank analogues for GDEPT when bystander activity is a key component of selection, and that the lipophilicity of the compound plays a major role. Pre-prodrug 6-P was ultimately identified as the most efficacious prodrug for NfsA_Ec, producing a tumor growth delay of 156% (*p* = 0.005) compared to untreated control tumors. A finding consistent with prodrug 6 having optimal ‘lipophilic efficiency’, a factor that has received significant attention in drug design and is perhaps best viewed as a selectivity index between potency and LogD_7.4_ [35].

A similar disconnect between the 2D and 3D SAR was observed for activation by AKR1C3. Whilst the 2D anti-proliferative screening indicated that all prodrugs appeared to be AKR1C3-resistant, in fact, there were only three bono fide AKR1C3-negative prodrugs in 3D MCL assays; compounds **1**, **2**, and **6**. Protein X-ray crystallography studies have previously indicated that the active site of AKR1C3 is significantly hydrophobic and can accommodate a range of lipophilic structures [36,37]. In the present series, a threshold LogD_7.4_ of approximately 1.6 appeared to be the cut-off after which AKR1C3 activation occurred. This is somewhat surprising, as the enlargement in steric bulk that is increasing the LogD_7.4_ should also, in theory, be causing increased interference with the NADPH co-factor in the binding pocket of AKR1C3. Nevertheless, we expect that removal of AKR1C3 activity will reduce or completely eliminate the large differences in toxicity and poor allometric scaling between species that was observed for PR-104 [21], potentially improving the MTD of these novel analogues in human patients. Studies in the appropriate pre-clinical animal models will be required to confirm this [22].

Finally, we confirmed that the lead compound **6** (and its phosphate pre-prodrug 6-P) retained hypoxia selectivity in vitro and in vivo (as a single agent and in combination with radiotherapy) at a comparable level to PR-104A and PR-104 when given at the HED of 388 μmol/kg (Figure 6). Areas of tumor hypoxia are an impediment to the efficacy of most cancer therapies. Hypoxic cells are resistant to radiation [38]; drug penetration into hypoxic tissue is poor [39]; and tumor hypoxia generates an immunosuppressive environment, thus inhibiting the anti-tumor immune response [40]. In addition, hypoxic stress can inhibit the RNA, DNA, and protein synthesis that is crucial for the replication of particular viral vectors, some of which are commonly employed in GDEPT regimens [41]. For example, hypoxia inhibits adenoviral replication by reducing translation of the important viral proteins E1a and fiber [42]. We hypothesize that a GDEPT strategy consisting of a replicating biological vector and a prodrug with dual NTR/hypoxic activation is an advantage over NTR-selective prodrug strategies, as there is potential for the vector to provide localized immune-stimulation in the tumor microenvironment in addition to elimination of the hypoxic compartment by the prodrug. This has the potential to synergize with a number of well-established cancer therapies to improve response. We conclude that compound **6** (when administered as 6-P), featuring an ethylsulfone moiety and an *N*-methylcarboxamide linker substituent, is an optimized dual NTR/hypoxia-activated prodrug that achieves a balance of maximized bystander activity without activation by AKR1C3. The structural features present in compound **6** further serve to impart excellent dose tolerance to pre-prodrug 6-P such that the combination of a high MTD and maximal bystander cell killing result in significant in vivo efficacy, recommending 6/6-P for advanced evaluation.

## 4. Materials and Methods

### 4.1. Chemical Synthesis

Elemental analyses were performed by the Campbell Microanalytical Laboratory, University of Otago, Dunedin, New Zealand. Melting points were determined using an Electrothermal IA9100 melting point apparatus and are as read. ^1^H NMR spectra were measured on a Bruker Avance 400 spectrometer at 400 MHz and were referenced to Me_4_Si or solvent resonances. Chemical shifts and coupling constants were recorded in units of ppm and hertz, respectively. High-resolution electrospray ionization (HR-ESI-MS) mass spectra were determined on a Bruker micrOTOF-Q II mass spectrometer or an Agilent 6530 Q-TOF mass spectrometer coupled to an Agilent 1200 series HPLC system. Liquid chromatography–mass spectrometry (LC–MS) was performed either on an Agilent 1100 LC system interfaced with an Agilent MSD mass detector or on a Micromass Platform LC mass spectrometer. In both cases, mass detection was performed with an APCI source through using simultaneous positive and negative ion acquisition. Column chromatography was performed on silica gel (Merck 230–400 mesh) using the indicated eluents. Thin-layer chromatography was carried out on aluminum-backed silica gel plates (Merck 60 F254), with visualization of components by UV light (254 nm), I_2_, or KMnO_4_ staining. Tested compounds (including batches screened in vivo) were 95% pure, as determined by elemental analysis and/or by HPLC conducted on an Agilent 1100 system with diode array detection, using a 150 mm × 3.2 mm Altima 5 mm reversed-phase C8 or C18 column. Elemental analyses indicated by the symbols of the elements were within ±0.4% of the theoretical values. Compounds **11**, **14**, and **17** were prepared as previously published [23]. The preparation of compound **1** by Method 1 and its elaboration to 1-P, via intermediate 45, has been previously reported [22].

#### 4.1.1. Synthesis of Compound **2**—Method 1

5-(Bis(2-chloroethyl)amino)-*N*-(2-hydroxyethyl)-*N*-methyl-4-(methylsulfonyl)-2-nitrobenzamide (**22**). A stirred solution of compound **20** [22] (1.00 g, 2.87 mmol) in SOCl_2_ (25 mL) was treated with DMF (3 drops) and heated under reflux for 4 h. The excess SOCl_2_ was removed by distillation under reduced pressure and the residue dissolved in CH_2_Cl_2_ (10 mL) and THF (6 mL), cooled to 0 °C, and treated with 2-(methylamino)ethanol (824 µL, 10.3 mmol). The reaction mixture was stirred at 0 °C for 20 min, then warmed to room temperature, acidified with aqueous HCl (0.5 M, 8 mL), and extracted with EtOAc (2×). The combined organic phases were washed with brine, dried with Na_2_SO_4_, and evaporated to dryness under reduce pressure. The crude product was purified by flash column chromatography on silica gel eluting with CH_2_Cl_2_/MeOH (25:1). The product obtained was triturated with EtOAc/hexanes to give the title compound **22** as a mixture of atropisomers and a yellow solid (395 mg, 31%), M.p. 142–144 °C. ^1^H NMR [(CD_3_)_2_SO] δ 8.66 (s, 0.4 H), 8.64 (s, 0.6 H), 7.71 (s, 0.6 H), 7.66 (s, 0.4 H), 4.83–4.78 (m, 1 H), 3.78 (br s, 4 H), 3.76–3.71 (m, 4 H), 3.69–3.64 (m, 1 H), 3.55–3.52 (m, 2 H), 3.48–3.47 (2s, 3 H), 3.19 (br s, 1 H), 3.04 (s, 1.6 H), 2.85 (s, 1.4 H). HRMS: calcd for C_15_H_21_Cl_2_N_3_NaO_6_S ([M + Na]^+^) 464.0407, found 464.0420.

5-(Bis(2-bromoethyl)amino)-*N*-(2-hydroxyethyl)-*N*-methyl-4-(methylsulfonyl)-2-nitrobenzamide (**2**). A solution of compound **22** (320 mg, 0.72 mmol) in 3-methyl-2-butanone (15 mL) was treated with LiBr (1.26 g, 14.5 mmol) and heated to reflux overnight. The reaction mixture was cooled to room temperature, and the solvent was removed under reduced pressure. The residue was dissolved in EtOAc and washed with water (3×), dried with Na_2_SO_4_, and concentrated under reduced pressure. The crude mixture was resubmitted to LiBr (2×) and worked up as above. The residue was purified by flash column chromatography on silica gel eluting with CH_2_Cl_2_/MeOH (20:1). The product obtained was triturated with EtOAc/hexanes to provide the title compound **2** as a mixture of atropisomers and a yellow solid (258 mg, 67%), M.p. 138–140 °C. ^1^H NMR [(CD_3_)_2_SO] δ 8.65 (s, 0.4 H), 8.64 (s, 0.6 H), 7.72 (s, 0.6 H), 7.67 (s, 0.4 H), 4.82–4.77 (m, 1 H), 3.84–3.77 (m, 4 H), 3.69–3.59 (m, 5 H), 3.58–3.52 (m, 1 H), 3.49–3.48 (2s, 3 H), 3.45–3.35 (br s, 1 H), 3.19 (br s, 1 H), 3.04 (s, 1.6 H), 2.86 (s, 1.4 H). APCI MS: 532.2 ([M + H]^+^). C_15_H_21_Br_2_N_3_O_6_S.^1^/_4_EtOAc (calculated): C = 34.74; H = 4.19; N = 7.60; observed: C = 35.06; H = 4.09; N = 7.64.

The synthesis of compounds **3** and **4** of Scheme 1 by Method 1 is described in the Supplementary Data.

#### 4.1.2. Synthesis of Compound **1**—Method 2

*tert*-Butyl 5-fluoro-4-(methylsulfonyl)-2-nitrobenzoate (**25**). Compound **17** (8.23 g, 31.3 mmol) was dissolved in CH_3_CN (48 mL) and treated with *tert*-butyl acetate (48 mL) and perchloric acid (70%, 2.64 mL, 43.8 mmol). The reaction mixture was stirred at room temperature for 48 h, and the solvents were removed under reduced pressure. The residue was recrystallized from MeOH/H_2_O at 6 °C overnight. The product was collected by filtration to provide the title compound **25** as pale-yellow crystals (5.71 g, 57%), M.p. 99–101 °C. ^1^H NMR (CDCl_3_) δ 8.58 (d, *J* = 5.7 Hz, 1 H), 7.57 (d, *J* = 8.5 Hz, 1 H), 3.30 (s, 3 H), 1.60 (s, 9 H). C_12_H_14_FNO_6_S (calculated): C = 45.14; H = 4.42; N = 4.39; observed: C = 45.44; H = 4.47; N = 4.32. Note: The filtrate was diluted with water and treated with aqueous HCl (4M) to precipitate unreacted starting material, which was collected by filtration.

*tert*-Butyl 5-(bis(2-hydroxyethyl)amino)-4-(methylsulfonyl)-2-nitrobenzoate (**28**). A solution of compound **25** (6.64 g, 20.8 mmol) in DMSO (15 mL) was treated with diethanolamine (2.81 mL, 29.1 mmol). The reaction mixture was stirred at room temperature for 2 h, then poured into a beaker of ice-water, extracted with diethyl ether (3×), dried with Na_2_SO_4_, and concentrated under reduced pressure. The crude product was purified by flash column chromatography on silica gel eluting with CH_2_Cl_2_/MeOH (19:1) to give the title compound **28** as a yellow gum (7.35 g, 87%). ^1^H NMR [(CD_3_)_2_SO] δ 8.50 (s, 1 H), 7.63 (s, 1 H), 4.64 (t, *J* = 5.0 Hz, 2 H), 3.58–3.54 (m, 4 H), 3.52–3.50 (m, 4 H), 3.45 (s, 3 H), 1.53 (s, 9 H). HRMS: calcd for C_16_H_25_N_2_O_8_S ([M + H]^+^) 405.1347, found 405.1326.

*tert*-Butyl 5-(bis(2-((methylsulfonyl)oxy)ethyl)amino)-4-(methylsulfonyl)-2-nitrobenzoate (**31**). A solution of compound **28** (8.52 g, 21.1 mmol) in CH_2_Cl_2_ (290 mL) was treated with Et_3_N (10.3 mL, 73.8 mmol), cooled to 0 °C, and treated with MsCl (4.90 mL, 63.2 mmol) by dropwise addition. The reaction mixture was stirred for 20 min at 0 °C, then warmed to room temperature, diluted with CH_2_Cl_2_, washed with water (3×), dried with Na_2_SO_4_, and concentrated under reduced pressure. The residue was purified by flash column chromatography on silica gel eluting with CH_2_Cl_2_/MeOH (19:1) to give the title compound **31** as a yellow gum (10.6 g, 90%). ^1^H NMR [(CD_3_)_2_SO] δ 8.52 (s, 1 H), 7.83 (s, 1 H), 4.36 (t, *J* = 5.2 Hz, 4 H), 3.77 (t, *J* = 5.0 Hz, 4 H), 3.43 (s, 3 H), 3.14 (s, 6 H), 1.53 (s, 9 H). HRMS: calcd for C_18_H_28_N_2_NaO_12_S_3_ ([M + Na]^+^) 583.0674, found 583.0697.

5-(Bis(2-((methylsulfonyl)oxy)ethyl)amino)-4-(methylsulfonyl)-2-nitrobenzoic acid (**34**). A solution of compound **31** (10.6 g, 18.9 mmol) in CH_2_Cl_2_ (56 mL) was treated with TFA (21 mL) at 5 °C. The reaction mixture was stirred at room temperature for 2 h, and the solvent and excess TFA were removed under reduced pressure. The residue was dissolved in EtOAc, and the solvent was evaporated to dryness to remove trace amounts of TFA. The residue was dried in a vacuum oven at 45 °C overnight to afford the title compound **34** as a yellow gum (9.56 g, quant.). ^1^H NMR [(CD_3_)_2_SO] δ 8.50 (s, 1 H), 7.89 (s, 1 H), 4.35 (t, *J* = 5.1 Hz, 4 H), 3.75 (t, *J* = 5.2 Hz, 4 H), 3.44 (s, 3 H), 3.14 (s, 6 H). HRMS: calcd for C_14_H_20_N_2_NaO_12_S_3_ ([M + Na]^+^) 527.0062, found 527.0071.

((5-((2-Hydroxyethyl)carbamoyl)-2-(methylsulfonyl)-4-nitrophenyl)azanediyl)bis(ethane-2,1-diyl) dimethanesulfonate (**37**). A solution of compound **34** (3.35 g, 6.64 mmol) in CH_2_Cl_2_ (100 mL) and CH_3_CN (25 mL) was treated with MgO (4.01 g, 99.6 mmol) at room temperature, then cooled to 0 °C and treated with oxalyl chloride (3.42 mL, 39.8 mmol) and DMF (3 drops). The reaction mixture was stirred at 0 °C for 1 h and warmed to room temperature for 3 h. The mixture was filtered through a short pad of Celite, and the solvents were removed under reduced pressure. The residue was dissolved in CH_2_Cl_2_ (100 mL) and THF (25 mL), cooled to 0 °C, treated with 2-aminoethanol (663 µL, 11.0 mmol), and warmed to room temperature for 20 min. The mixture was washed with water (3×), dried with Na_2_SO_4_, and concentrated under reduced pressure. The residue was purified by flash column chromatography on silica gel eluting with CH_2_Cl_2_/MeOH (19:1) to afford the title compound **37** as a yellow gum (3.42 g, 94%). ^1^H NMR [(CD_3_)_2_SO] δ 8.71 (t, *J* = 5.7 Hz, 1 H), 8.51 (s, 1 H), 7.73 (s, 1 H), 4.77 (t, *J* = 5.0 Hz, 1 H), 4.35 (t, *J* = 5.2 Hz, 4 H), 3.72 (t, *J* = 5.2 Hz, 4 H), 3.55–3.51 (m, 2 H), 3.44 (s, 3 H), 3.34–3.29 (m, 2 H), 3.16 (s, 6 H). HRMS: calcd for C_16_H_25_N_3_NaO_12_S_3_ ([M + Na]^+^) 570.0497, found 570.0493.

5-(Bis(2-bromoethyl)amino)-*N*-(2-hydroxyethyl)-4-(methylsulfonyl)-2-nitrobenzamide (**1**). Compound **37** (3.40 g, 6.21 mmol) was dissolved in acetone (180 mL) and treated with LiBr (10.8 g, 124.2 mmol) at room temperature. The reaction mixture was stirred overnight, and the solvent was removed. The residue was dissolved in EtOAc and washed with water (2×), dried with Na_2_SO_4_, and concentrated under reduced pressure. The crude product was purified by flash column chromatography on silica gel eluting with CH_2_Cl_2_/MeOH (20:1) and further recrystallized from CH_2_Cl_2_/MeOH (4:1) and iPr_2_O to give 1 as a pale-yellow solid (3.06 g, 95%). ^1^H NMR identical to that described previously [22].

#### 4.1.3. Synthesis of Compounds **5** and **6**—Method 2

4-(Ethylsulfonyl)-3-fluorobenzaldehyde (**12**). 3,4-Difluorobenzaldehyde 10 (23.5 g, 165.4 mmol) was treated with sodium ethanesulfinate (23.1 g, 198.8 mmol) in DMSO (230 mL) at room temperature. The reaction mixture was heated at 75 °C under N_2_ for 3 h, then cooled to room temperature and poured into a beaker of ice-water. The solid was collected by filtration, washed with water, and dried in a vacuum oven at 45 °C overnight. The crude product was purified by flash column chromatography on silica gel eluting with CH_2_Cl_2_/hexane (4:1) then neat CH_2_Cl_2_ to provide the title compound **12** as a white solid (28.7 g, 80%), M.p. 107–110 °C. ^1^H NMR (CDCl_3_) δ 10.09 (d, *J* = 1.9 Hz, 1 H), 8.16 (dd, *J* = 6.5, 1.4 Hz, 1 H), 7.87 (dd, *J* = 7.9, 1.4 Hz, 1 H), 7.75 (dd, *J* = 9.4, 1.4 Hz, 1 H), 3.38 (q, *J* = 7.4 Hz, 2 H), 1.33 (t, *J* = 7.5 Hz, 3 H). C_9_H_9_FO_3_S (calculated): C = 49.99; H = 4.20; F = 8.79; observed: C = 50.19; H = 4.23; F = 8.91.

4-(Ethylsulfonyl)-3-fluorobenzoic acid (**15**). A solution of compound **12** (30.7 g, 141.9 mmol) in CH_3_CN (280 mL) was treated with a buffer solution of NaH_2_PO_4_.4H_2_O (4.81 g, 30.8 mmol) and conc. HCl (3 mL) in water (105 mL), then H_2_O_2_ (35%, 24 mL, 709 mmol) at room temperature. The reaction mixture was cooled to 0 °C and treated with a solution of NaClO_2_ (18.0 g, 199 mmol) in water (350 mL) by dropwise addition. After stirring at room temperature for 5 h, the solvents were concentrated to half a volume under reduced pressure, and the solid was collected by filtration. The filtrate was treated with conc. HCl to precipitate further product that was collected by filtration. The combined solid was dried in a vacuum oven at 45 °C overnight to give the title compound **15** as a white solid (33.0 g, 99%), M.p. 184–186 °C. ^1^H NMR [(CD_3_)_2_SO] δ 13.81 (br s, 1 H), 8.01–7.98 (m, 2 H), 7.96–7.91 (m, 1 H), 3.45 (q, *J* = 7.4 Hz, 2 H), 1.52 (t, *J* = 7.3 Hz, 3 H). APCI MS: 231.2 ([M–H]^-^) C_9_H_9_FO_4_S (calculated): C = 46.55; H = 3.91; F = 8.18; observed: C = 46.82; H = 3.99; F = 8.33.

4-(Ethylsulfonyl)-5-fluoro-2-nitrobenzoic acid (**18**). Compound **15** (15.0 g, 64.7 mmol) was dissolved in conc. H_2_SO_4_ (107 mL) and treated with fuming HNO_3_ (21 mL) by dropwise addition at room temperature. The reaction mixture was heated at 45 °C for 4 h, cooled to room temperature, and poured into a beaker of ice-water. The solid was collected by filtration, washed several times with water, and dried in a vacuum oven at 45 °C overnight to afford the title compound **18** as a pale yellow solid (14.5 g, 81%), m.p. 140–143 °C. ^1^H NMR [(CD_3_)_2_SO] δ 14.59 (br s, 1 H), 8.41 (d, *J* = 5.8 Hz, 1 H), 8.07 (d, *J* = 9.3 Hz, 1 H), 3.53 (q, *J* = 7.4 Hz, 2 H), 1.20 (t, *J* = 7.4 Hz, 3 H). HRMS: calcd for C_9_H_9_FNO_6_S ([M + H]^+^) 278.0130, found 278.0129.

*tert*-Butyl 4-(ethylsulfonyl)-5-fluoro-2-nitrobenzoate (**26**). Reaction of compound **18** (24.4 g, 87.8 mmol) with *tert*-butyl acetate (150 mL) and perchloric acid (70%, 3.70 mL, 61.4 mmol), using the same procedure as described for 25, afforded the title compound **26** as pale-yellow crystals (20.5 g, 70%), M.p. 105–107 °C. ^1^H NMR (CDCl_3_) δ 8.54 (d, *J* = 5.7 Hz, 1 H), 7.54 (d, *J* = 8.5 Hz, 1 H), 3.37 (q, *J* = 7.6 Hz, 2 H), 1.59 (s, 9 H), 1.37 (t, *J* = 7.4 Hz, 3 H). C_13_H_16_FNO_6_S.^1^/_10_iPr_2_O (calculated): C = 47.58; H = 5.05; N = 4.08; observed: C = 47.35; H = 4.87; N = 4.17.

*tert*-Butyl 5-(bis(2-hydroxyethyl)amino)-4-(ethylsulfonyl)-2-nitrobenzoate (**29**). Reaction of compound **26** (7.00 g, 21.0 mmol) with diethanolamine (2.80 mL, 29.4 mmol) in DMSO (10 mL), using the same procedure as described for 28, afforded the title compound 29 as a yellow gum (7.35 g, 84%). ^1^H NMR [(CD_3_)_2_SO] δ 8.48 (s, 1 H), 7.64 (s, 1 H), 4.64 (t, *J* = 4.8 Hz, 2 H), 3.71 (q, *J* = 7.3, 2 H), 3.57–3.49 (m, 8 H), 1.53 (s, 9 H), 1.00 (t, *J* = 7.3 Hz, 3 H). HRMS: calcd for C_17_H_27_N_2_O_8_S ([M + H]^+^) 419.1483, found 419.1483.

*tert*-Butyl 5-(bis(2-((methylsulfonyl)oxy)ethyl)amino)-4-(ethylsulfonyl)-2-nitrobenzoate (**32**). Reaction of compound **29** (7.35 g, 17.6 mmol) with MsCl (4.08 mL, 52.7 mmol) and Et_3_N (8.56 mL, 61.4 mmol) in DCM (280 mL), using the same procedure as described for 31, provided the title compound **32** as a yellow gum (9.40 g, 93%). ^1^H NMR [(CD_3_)_2_SO] δ 8.50 (s, 1 H), 7.81 (s, 1 H), 4.36 (t, *J* = 5.0 Hz, 4 H), 3.77 (t, *J* = 5.0 Hz, 4 H), 3.64 (q, *J* = 7.4 Hz, 2 H), 3.15 (s, 6 H), 1.53 (s, 9 H), 1.08 (t, *J* = 7.3 Hz, 3 H). HRMS: calcd for C_19_H_31_N_2_O_12_S_3_ ([M + H]^+^) 575.1020, found 575.1034.

5-(Bis(2-((methylsulfonyl)oxy)ethyl)amino)-4-(ethylsulfonyl)-2-nitrobenzoic acid (**35**). Reaction of compound **32** (9.40 g, 16.4 mmol) with TFA (27 mL) in DCM (54 mL), using the same procedure as described for 34, afforded the title compound **35** as a yellow gum (8.73 g, 99%). ^1^H NMR [(CD_3_)_2_SO] δ 8.50 (s, 1 H), 7.88 (s, 1 H), 4.35 (t, *J* = 5.0 Hz, 4 H), 3.75 (t, *J* = 5.0 Hz, 4 H), 3.65 (q, *J* = 7.3 Hz, 2 H), 3.15 (s, 6 H), 1.07 (t, J = 7.4 Hz, 3 H). HRMS: calcd for C_15_H_23_N_2_O_12_S_3_ ([M + H]^+^) 519.0413, found 519.0408.

((2-(Ethylsulfonyl)-5-((2-hydroxyethyl)carbamoyl)-4-nitrophenyl)azanediyl)bis(ethane-2,1-diyl) dimethanesulfonate (**40**). Reaction of the freshly prepared acid chloride of compound **35** (4.31 g, 8.02 mmol) with 2-aminoethanol (970 μL, 16.0 mmol), in DCM/THF (2:1 ratio, 105 mL), using the same procedure as described for 37, provided the title compound **40** as a yellow gum (2.71 g, 60%). ^1^H NMR [(CD_3_)_2_SO] δ 8.69 (t, *J* = 5.6 Hz, 1 H), 8.49 (s, 1 H), 7.70 (s, 1 H), 4.77 (t, *J* = 5.4 Hz, 1 H), 4.35 (t, *J* = 5.1 Hz, 4 H), 3.73 (t, *J* = 5.1 Hz, 4 H), 3.65 (q, *J* = 7.4 Hz, 2 H), 3.53 (q, *J* = 6.2 Hz, 2 H), 3.31 (t, *J* = 6.0 Hz, 2 H), 3.17 (s, 6 H), 1.05 (t, *J* = 7.3 Hz, 3 H). HRMS: calcd for C_17_H_28_N_3_O_12_S_3_ ([M + H]^+^) 562.0837, found 562.0830.

5-(Bis(2-bromoethyl)amino)-*N*-(2-hydroxyethyl)-4-(ethylsulfonyl)-2-nitrobenzamide (**5**). Reaction of compound **40** (2.71 g, 4.83 mmol) with LiBr (8.40 g, 96.5 mmol) in acetone (150 mL), using the same procedure as described for 1 (Method 2), afforded 5 as a yellow solid (2.32 g, 91%), M.p. 137–140 °C. ^1^H NMR [(CD_3_)_2_SO] δ 8.75 (t, *J* = 5.6 Hz, 1 H), 8.50 (s, 1 H), 7.67 (s, 1 H), 4.78 (t, *J* = 5.4 Hz, 1 H), 3.76 (t, *J* = 6.9 Hz, 4 H), 3.71 (q, *J* = 7.4 Hz, 2 H), 3.62 (t, *J* = 6.8 Hz, 4 H), 3.54 (q, *J* = 6.2 Hz, 2 H), 3.31 (t, *J* = 6.0 Hz, 2 H), 1.07 (t, *J* = 7.3 Hz, 3 H). APCI MS: 532.2 ([M + H]^+^). C_15_H_21_Br_2_N_3_O_6_S (calculated): C = 33.91; H = 3.98; N = 7.91; observed: C = 34.2; H = 3.93; N = 7.73.

((5-((2-Hydroxyethyl)(methyl)carbamoyl)-2-(ethylsulfonyl)-4-nitrophenyl)azanediyl)bis(ethane-2,1-diyl) dimethanesulfonate (**41**). Reaction of the freshly prepared acid chloride of compound **35** (4.48 g, 8.34 mmol) with 2-(methylamino)ethanol (1.34 mL, 16.7 mmol) in DCM/THF (2:1 ratio, 105 mL), using the same procedure as described for 37, afforded the title compound **41** as a mixture of atropisomers and a yellow gum (3.17g, 66%). ^1^H NMR [(CD_3_)_2_SO] δ 8.64 (s, 0.5 H), 8.62 (s, 0.5 H), 7.71 (s, 0.5 H), 7.65 (s, 0.5 H), 4.84–4.81 (m, 1 H), 4.36–4.33 (m, 4 H), 3.79–3.76 (m, 2 H), 3.73 (br s, 2 H), 3.66–3.53 (m, 5 H), 3.20 (br s, 1 H), 3.15 (s, 6 H), 3.04 (s, 1.5 H), 2.86 (s, 1.5 H), 1.10 (t, *J* = 7.4 Hz, 3 H). HRMS: calcd for C_18_H_29_N_3_NaO_12_S_3_ ([M + Na]^+^) 598.0794, found 598.0809.

5-(Bis(2-bromoethyl)amino)-*N*-(2-hydroxyethyl)-*N*-methyl-4-(ethylsulfonyl)-2-nitrobenzamide (**6**). Reaction of compound **41** (3.05 g, 5.30 mmol) with LiBr (9.22 g, 106 mmol) using the same procedure as described for 1 (Method 2), provided 6 as a mixture of atropisomers and a yellow gum (2.46 g, 85%). ^1^H NMR [(CD_3_)_2_SO] δ 8.64 (s, 0.4 H), 8.63 (s, 0.6 H), 7.70 (s, 0.6 H), 7.66 (s, 0.4 H), 4.81 (t, *J* = 5.5 Hz, 0.4 H), 4.78 (t, *J* = 5.5 Hz, 0.6 H), 3.84–3.76 (m, 4 H), 3.73–3.66 (m, 3 H), 3.64–3.58 (m, 4 H), 3.55–3.52 (m, 1 H), 3.18 (br s, 2 H), 3.04 (s, 1.6 H), 2.86 (s, 1.4 H), 1.10 (t, J = 7.4 Hz, 3 H). HRMS: calcd for C_16_H_23_Br_2_N_3_NaO_6_S ([M + Na]^+^) 565.9556, found 565.9567.

The synthesis of compounds **3**, **4**, and **7**–**9** of Scheme 1 by Method 2 is described in the Supplementary Data.

#### 4.1.4. Synthesis of Compound 2-P

2-(5-(Bis(2-bromoethyl)amino)-*N*-methyl-4-(methylsulfonyl)-2-nitrobenzamido)ethyl di-*tert*-butyl phosphate (**46**). To a solution of compound **2** (3.13 g, 5.88 mmol) in DMF (4 mL) at 5 °C, we added a 1*H*-tetrazole solution (3% in CH_3_CN, 63 mL, 27.1 mmol), followed by di-*tert*-butyl-*N*,*N*-diisopropylphosphoramidite (7.4 mL, 23.5 mmol). The reaction mixture was stirred for 4 h at room temperature, diluted with CH_2_Cl_2_ (25 mL), and cooled to 0 °C before solid *m*-CPBA (70%, 7.77 g, 44.1 mmol) was added portion-wise. The mixture was warmed to room temperature, stirred for a further 1 h, and then the solvents were removed under reduced pressure. The residue was dissolved in EtOAc, washed with a 10% solution of sodium disulfite (2×) and then a 5% solution of sodium bicarbonate (3×), dried with Na_2_SO_4_, and concentrated under reduced pressure. The crude product was purified by flash column chromatography on silica gel eluting with CH_2_Cl_2_/MeOH (25:1) to give the title compound **46** as a mixture of atropisomers and a yellow gum (3.23 g, 76%). ^1^H NMR [(CD_3_)_2_SO] δ 8.67 (s, 0.5 H), 8.66 (s, 0.5 H), 7.78 (s, 0.5 H), 7.60 (s, 0.5 H), 4.15–4.02 (m, 2 H), 3.85–3.81 (m, 4 H), 3.78–3.66 (m, 2 H), 3.64–3.61 (m, 4 H), 3.49 (s, 3 H), 3.07 (s, 1.5 H), 2.89 (s, 1.5 H), 1.44 (s, 10 H), 1.40 (s, 8 H). HRMS: calcd for C_23_H_38_Br_2_N_3_NaO_9_PS ([M + Na]^+^) 744.0301, found 744.0325.

2-(5-(Bis(2-bromoethyl)amino)-*N*-methyl-4-(methylsulfonyl)-2-nitrobenzamido)ethyl dihydrogen phosphate (2-P). Compound **46** (3.23 g, 4.46 mmol) in CH_2_Cl_2_ (17 mL) was cooled to 5 °C and treated with TFA (17 mL). The reaction mixture was stirred for 1 h at room temperature, and the solvent and the excess TFA were removed under reduced pressure. The residue was triturated with CH_2_Cl_2_/iPr_2_O, then dissolved in CH_3_CN. The solvent was removed under reduced pressure to provide 2-P as a mixture of atropisomers and a yellow gum (2.72 g, quant.). ^1^H NMR [(CD_3_)_2_SO] δ 8.66 (s, 0.5 H), 8.65 (s, 0.5 H), 7.78 (s, 0.5 H), 7.63 (s, 0.5 H), 4.11–4.06 (m, 2 H), 3.84–3.81 (m, 4 H), 3.78–3.65 (m, 2 H), 3.61–3.58 (m, 4 H), 3.46 (s, 3 H), 3.04 (s, 1.5 H), 2.86 (s, 1.5 H). HRMS: calcd for C_15_H_22_Br_2_N_3_NaO_9_PS ([M + Na] ^+^) 631.9065, found 631.9073.

The synthesis of compound 6-P of Scheme 2 is described in the Supplementary Data.

### 4.2. Drug Information and Formulation

PR-104A was synthesized, purified, and stored as previously reported [43,44]. PR-104 was supplied by Proacta Incorporated (La Jolla, CA, USA). For in vitro studies, prodrug stocks were dissolved in DMSO (0.1 M) and stored at −80 °C. For in vivo studies, PR-104 (PR-104 sodium lyophilized with mannitol) was reconstituted in 2 mL water and diluted in phosphate-buffered saline (PBS). 1-P, 2-P, and 6-P free acids were dissolved in phosphate-buffered saline containing two equivalents of NaHCO_3_. Dosing solutions were prepared fresh, held a room temperature in amber vials, and were used within three hours.

### 4.3. Determination of LogD_7.4_

Distribution ratios (LogD_7.4_) were determined by a low-volume octanol/PBS shake-flask method [45,46,47]. Briefly, saturated solutions of octanol and PBS were prepared by mixing equal volumes of n-octanol and PBS (pH 7.4) before being left for three days. The octanol and PBS phases were then carefully separated and left to stand for at least 24 h. Stock solutions (0.1–0.5 mM) of each compound were prepared in octanol saturated with PBS. The stock solutions were diluted with octanol saturated with PBS containing and inter-assay control (acridone) to a final compound concentration of 50 μM. Aliquots (450 μL) were then taken in triplicate, and 450 μL of PBS saturated with octanol was added into amber tubes. The tubes were then sealed in parafilm and rotated for 90 min at 30 rpm to equilibrate. After centrifugation (15,000× *g*, 5 min, 20°C) the octanol and PBS phases were separated into different tubes and the octanol phase diluted with methanol (1:5 *v*/*v*). Both phases were analyzed by HPLC (diode-array detector at 364 ± 10 nm) by injecting 50 μL of the PBS phase and 15 μL of the diluted octanol phase. The results were then expressed as the log ratio of the peak areas obtained from the octanol and PBS phases corrected for dilution and injection volume.

### 4.4. Cell Lines and Candidate Gene Expression

HCT116 WT cells were purchased from the ATCC (Manassas, VA, USA). HCT116 cell lines over-expressing AKR1C3 [48] and NfsA_Ec [15] had been previously generated and validated for candidate gene expression as described. SiHa cells were a generous gift from Dr David Cowan (Ontario Cancer Institute). H1299 WT cells were purchased from Onyx Pharmaceuticals (South San Francisco, CA, USA). H1299 cells over-expressing NfsA_Ec were previously generated and validated as described [16]. STR phenotyping confirmed authenticity for each cell line. Frozen stocks were confirmed to be mycoplasma free by PCR enzyme-linked immunosorbent assay (ELISA) (Roche Diagnostics Corp, Basel, Switzerland). Neoplastic cell lines were cultured in α-minimal essential medium using a humidified incubator (37 °C, 5% CO_2_) as previously described [14].

### 4.5. Anti-Proliferative (IC_50_) Assays

The anti-proliferative IC_50_ was determined as the concentration of prodrug required for 50% inhibition of cell growth, following drug exposure for four hours, then five days regrowth in the absence of drug. Assays were performed under oxic or anoxic conditions as described previously [14,49], the latter by using a 5% H_2_/palladium catalyst scrubbed Bactron anaerobic chamber (Sheldon Manufacturing, Cornelius, OR, USA) to achieve severe anoxia (<10 ppm O_2_ gas phase) during prodrug exposure. Total exposure to anoxia did not exceed 6 h.

### 4.6. Multicellular Layer Assays

Multicellular layers (MCLs) were grown, and bystander effect assays were performed as previously reported [4].

### 4.7. Animal Husbandry

Specific pathogen-free homozygous NIH-III (NIH-Lystbg Foxn1nu Btkxid) nude mice were obtained from Charles River Laboratories (Wilmington, MA, USA), bred in Vernon Jansen Unit (shared vivarium, University of Auckland), and supplied at 7–9 weeks of age. Mice were housed in groups of ≤6 in Techniplast microisolator cages with a 12 h light/dark cycle and were fed a standard rodent diet (Harlan Teklad diet 2018i) and water ad libitum. All animals were uniquely identifiable by ear tag number and weighed 18 to 25 g at the time of the experiment. All animal protocols were approved by the University of Auckland Animal Ethics Committee (approval CR830).

### 4.8. Tumor Growth Delay Assay

The maximum tolerated doses of 1-P, 2-P, and 6-P were determined in tumor-free and H1299 tumor-bearing NIH-III nude mice using a fixed 8-step logarithmic scale with 1.33-fold dose increments as required. An estimated starting dose was determined from related compounds. Animals were promptly euthanized if body weight loss exceeded 20% or if there were clinical signs of severe morbidity. The MTD was defined as the highest dose, at which all animals survived (death rate < 1/6) with no unacceptable morbidity, and where the next dose increment had caused at least one death. Tumors were inoculated onto the flanks of NIH-III nude mice by subcutaneous injection of 10^7^ H1299 cells. Tumor-bearing mice were randomized to treatment groups (n = 5 mice per group) when tumors reached treatment size (300–350 mm^3^) and injected with a single intraperitoneal dose of pre-prodrug. Tumor size and body weights were measured regularly. Tumor volume was calculated as π(Lxw^2^)/6, where L is the major axis and w is the perpendicular minor axis. Animals were euthanized when the tumor volume had increased fourfold relative to pre-treatment volume (RTV4, survival endpoint) or if body weight loss exceeded 20% of the pre-treatment value. Kaplan–Meier plots were constructed, and median time to endpoint was calculated. Treatment efficacy was assessed by comparing the median survival time with untreated animals using the Log-rank test *p*-test (Sigmaplot version 11.0).

### 4.9. Tumor Excision Assay

When tumors reached treatment size (>400 mm^3^), mice were treated with drug by a single intraperitoneal injection of pre-prodrug or vehicle. Mice treated with ionizing radiation were exposed to 10 Gray prior to drug treatment, using a cobalt-60 source from an Eldorado G unit. Animals were euthanized 18 h after drug treatment, and tumors were excised and homogenized using sterile scissors until a fine mince was obtained. Following enzymatic dissociation (2.5 mg/mL pronase, 1 mg/mL collagenase, and 0.2 mg/mL DNAase in αMEM + 10% FBS + 1% P/S, 1 mL per 50 mg), cells were plated at concentrations of 10^2^–10^5^ cells per 60 mm dish and grown for 10 days before colonies were stained with methylene blue (2 g/L in 50% aqueous alcohol). Colonies containing >50 cells were counted as clonogenic survivors. From these values, plating efficiency (colonies/cells plated), number of clonogens per gram of tumor tissue (plating efficiency × total cells), and log cell kill relative to vehicle treated controls (log clonogens per gram) were calculated. Statistical analysis was undertaken using Holm–Sidak’s one-way ANOVA (Sigmaplot version 11.0).

### 4.10. Ex Vivo Phenotyping of Tumor Xenografts

The percentage of nitroreductase-expressing cells in the xenografts at the time of pre-prodrug treatment was determined using ex vivo pimonidazole flow cytometry on five untreated tumor homogenates as previously described [33]. Briefly, 1 × 10^6^ cells from each homogenate were seeded in a 6-well plate and exposed to 20 μM pimonidazole for two hours. Cells were then fixed in 4% paraformaldehyde in PBS for an hour at 4 °C. After fixation, cells were blocked in 10% BSA/PBS + 0.2% Tween-20 for an hour before exposure to the pimonidazole antibody (1:50 dilution, Hypoxyprobe-1 kit; Chemicon International, Temecula, CA, USA) for two hours. Cells were rinsed three times (2× PBS-Tween-20, 1× PBS) before fluorescence analysis was performed using a Becton Dickinson LSR-II flow cytometer.

## 5. Conclusions

In this study, we identified prodrug 6 as an optimal lipophilic nitrobenzamide mustard prodrug for activation by NfsA_Ec in a cancer gene therapy context when administered as pre-prodrug 6-P. Elimination of off-target AKR1C3 activity should allow for more accurate toxicokinetic scaling of the MTD from preclinical species to humans, and retention of hypoxia selectivity has the potential to provide additional therapeutic benefit. Of significant interest, interrogation of the SAR in this novel analogue series demonstrated that 2D monolayer assays can severely underestimate the potency of more lipophilic compounds, due to the presence of large extracellular dilution effects that presumably lead to cytotoxic metabolite ‘washout’ into excess media. For the development of compounds where the bystander effect plays an important role, high cell density assays where cytotoxic metabolites can diffuse into neighboring cells are critical to accurately determine therapeutic efficacy.

## 6. Patents

Patents arising from this work include the international PCT applications WO2005042471A1 and WO2014031012A1. The latter assigned to Health Innovation Ventures, leading to the issued patents EP2888227B1, US10202408B2, CA2886574C, US9873710B2, AU2013/306514B2, and US9505791B2.

## Figures and Tables

**Table 1:** Structures and lipophilicity of the mustard prodrug analogue series 1–9.

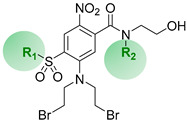			
**Cmpd**	**R_1_**	**R_2_**	**LogD_7.4_^a^**
**1**	Me	H	1.0
**2**	Me	Me	1.2
**3**	Me	Et	1.6
**4**	Me	*n*-Pr	2.1
**5**	Et	H	1.5
**6**	Et	Me	1.6
**7**	Et	Et	1.9 ^b^
**8**	*n*-Pr	H	1.8 ^b^
**9**	*n*-Pr	Me	2.1

^a^ Octanol-water partition coefficient, measured using a shake-flask method at pH 7.4. ^b^ Calculated using measured examples.

## Data Availability

Data is contained within the article and supplementary material.

## Supplementary Materials

The following are available online at www.mdpi.com/article/10.3390/ph15020185/s1, Synthesis of compounds **3** and **4** of Scheme 1 (Method 1). Synthesis of compounds **2**, **4**, **7**–**9** of Scheme 1 (Method 2). Synthesis of compound 6-P of Scheme 2. Figure S1: Correlation between lipophilicity (LogD_7.4_) and WT:NfsA_Ec IC_50_ ratio. Figure S2: Identification of alternative nitroreductases able to activate compound **6**. Table S1: Maximum tolerated dose of test compounds in NIH-III nude mice; study summary. Table S2: In vitro anti-proliferative activity of the three lead prodrug compounds in H1299 cells expressing *E. coli* NfsA, Table S3: Steady-state kinetic parameters for reduction of compound **6** and PR-104A by NfsA_Ec.
