# Supplementary material for: Interrogation of the Structure–Activity Relationship of a Lipophilic Nitroaromatic Prodrug Series Designed for Cancer Gene Therapy Applications
Source: Pharmaceuticals (Basel). 2022 Feb 1;15(2):185. doi: 10.3390/ph15020185 (PMC8877822; doi:10.3390/ph15020185)
Supplement: Supplementary file 1 [file pharmaceuticals-15-00185-s001.zip › pharmaceuticals-1534655-supplementary.pdf]

pharmaceuticals

Supplementary data

Interrogation of the Structure–Activity Relationship of a Lipophilic Nitroaromatic Prodrug Series Designed for Cancer Gene Therapy Applications

Amir Ashoorzadeh, Alexandra M. Mowday, Christopher P. Guise, Shevan Silva, Matthew R. Bull, Maria R. Abbattista, Janine N. Copp, Elsie M. Williams, David F. Ackerley, Adam V. Patterson and Jeff B. Smaill

Contents:

Synthesis of compounds 3 and 4 of Scheme 1 by Method 1.

Synthesis of compounds 2, 4, 7-9 of Scheme 1 by Method 2.

Synthesis of compound 6-P of Scheme 2.

Supplementary Figure S1. Correlation between lipophilicity ($LogD_{7.4}$) and WT:NfsA_Ec IC_{50} ratio.

Supplementary Figure S2. Identification of alternative nitroreductases able to activate compound **6**.

Supplementary Table S1. Summary of maximum tolerated dose of test compounds in NIH-III nude mice.

Supplementary Table S2. *In vitro* anti-proliferative activity of the three lead prodrug compounds in H1299 cells expressing *E. coli* NfsA.

Supplementary Table S3. Steady-state kinetic parameters for reduction of compound **6** and PR-104A by NfsA_Ec.

Synthesis of compounds 3 and 4 of Scheme 1 – Method 1

5-(Bis(2-chloroethyl)amino)-*N*-(2-hydroxyethyl)-*N*-ethyl-4-(methylsulfonyl)-2-nitrobenzamide (23). Reaction of the freshly prepared acid chloride of compound 20 (298 mg, 0.74 mmol) with 2-(ethylamino)ethanol (252 µL, 2.58 mmol) in THF (30 mL), using the same procedure as described for 22, afforded the title compound 23 as a mixture of atropisomers and a yellow solid (153 mg, 46%), M.p. 152–153 °C. ^{1}H NMR [$(CD_3)_2SO$] δ 8.66 (s, 0.4 H), 8.64 (s, 0.6 H), 7.69 (s, 1 H), 4.80 (t, *J* = 5.5 Hz, 0.4 H), 4.77 (t, *J* = 5.4 Hz, 0.6 H), 3.78–3.72 (m, 8 H), 3.67–3.66 (m, 1 H), 3.60–3.50 (br s, 2 H), 3.48–3.47 (2s, 3 H), 3.29–3.14 (m, 3 H), 1.20 (t, *J* = 7.1 Hz, 1.8 H), 1.02 (t, *J* = 7.1 Hz, 1.2 H). $C_{16}H_{23}Cl_2N_3O_6S.^1/_{10}$Hexanes (Calculated): C = 42.88; H = 5.29; Cl = 15.25; N = 9.04; observed: C = 42.85; H = 5.37; Cl = 15.00; N = 8.83.

5-(Bis(2-bromoethyl)amino)-*N*-(2-hydroxyethyl)-*N*-ethyl-4-(methylsulfonyl)-2-nitrobenzamide (3). Reaction of compound 23 (125 mg, 0.27 mmol) with LiBr (477 mg, 5.48 mmol) in 3-methyl-2-butanone (5 mL), using the same procedure as described for 2, afforded 3 as a mixture of atropisomers and a yellow solid (139 mg, 93%), M.p. 136-139 °C. ^{1}H NMR [$(CD_3)_2SO$] δ 8.66 (s, 0.4 H), 8.64 (s, 0.6 H), 7.71 (s, 0.4 H), 7.70 (s, 0.6 H), 4.80 (t, *J* = 5.0 Hz, 0.4 H), 4.76 (t, *J* = 4.9 Hz, 0.6 H), 3.84–3.76 (m, 4 H), 3.66–3.61 (m, 5 H), 3.50–3.49 (2s, 3 H), 3.44–3.36 (br s, 1 H), 3.29–3.16 (m, 4 H), 1.20 (t, *J* = 7.1 Hz, 1.8 H), 1.03 (t, *J* = 7.1 Hz, 1.2 H). HRMS calcd for $C_{16}H_{24}Br_2N_3O_6S$ ([M+H]$^+$) 545.9718, found 545.9725.

5-(Bis(2-chloroethyl)amino)-*N*-(2-hydroxyethyl)-*N*-propyl-4-(methylsulfonyl)-2-nitrobenzamide (24). Reaction of freshly prepared acid chloride of compound 20 (487 mg, 1.21 mmol) with 2-(propylamino)ethanol (484 µL, 4.23 mmol) in THF (50 mL), using the same procedure as described for 22, afforded the title compound 24 as a mixture of atropisomers and a yellow solid (182 mg, 32%), M.p. 158–161 °C. ^{1}H NMR [$(CD_3)_2SO$] δ 8.67 (s, 0.4 H), 8.64 (s, 0.6 H), 7.68 (s, 1 H), 4.80–4.76 (m, 1 H), 3.76–3.72 (m, 8 H), 3.67–3.66 (m, 1 H), 3.60–3.50 (br s, 1 H), 3.48 (s, 3 H), 3.17–3.08 (m, 4 H), 1.69–1.63 (m, 1 H), 1.51 (br s, 1 H), 0.94 (t, *J* = 7.4 Hz, 1.8 H), 0.68 (t, *J* = 7.4 Hz, 1.2 H). $C_{17}H_{25}Cl_2N_3O_6S.^1/_{10}$Hexanes (Calculated): C = 44.13; H = 5.56; N = 8.77; observed: C = 44.22; H = 5.72; N = 8.67.

5-(Bis(2-bromoethyl)amino)-*N*-(2-hydroxyethyl)-*N*-propyl-4-(methylsulfonyl)-2-nitrobenzamide (4). Reaction of compound 24 (182 mg, 0.39 mmol) with LiBr (674 mg, 7.75 mmol) in 3-methyl-2-butanone (8 mL), using the same procedure as described for 2, afforded 4 as a mixture of atropisomers and a yellow solid (130 mg, 60%), M.p. 154-156 °C. ^{1}H NMR [$(CD_3)_2SO$] δ 8.66 (s, 0.4 H), 8.64 (s, 0.6 H), 7.70 (s, 0.4 H), 7.69 (s, 0.6 H), 4.79–4.74 (m, 1 H), 3.83–3.76 (m, 4 H), 3.66–3.60 (m, 6 H), 3.49 (s, 3 H), 3.46–3.42 (br s, 1 H), 3.17–3.09 (m, 3 H), 1.69–1.64 (m, 1 H), 1.52 (br s, 1 H), 0.94 (t, *J* = 7.4 Hz, 1.8 H), 0.69 (t, *J* = 7.4 Hz, 1.2 H). APCI MS: 560.3 ([M + H]$^+$). $C_{17}H_{25}Br_2N_3O_6S.^1/_4$Hexanes (Calculated): C = 38.26; H = 4.95; N = 7.23; observed: C = 38.39; H = 4.64; N = 7.27.

Synthesis of compounds 2, 4, 7-9 of Scheme 1 – Method 2

((5-((2-Hydroxyethyl)(methyl)carbamoyl)-2-(methylsulfonyl)-4-nitrophenyl)azanediyl)bis(ethane-2,1-diyl) dimethanesulfonate (38). Reaction of the freshly prepared acid chloride of compound 34 (2.75 g, 5.27 mmol) with 2-(methylamino)ethanol (696 µL, 8.68 mmol) in DCM/THF (2:1 ratio, 75 mL), using the same procedure as described for 37, afforded the title compound 38 as a mixture of atropisomers and a yellow gum (2.22 g, 74%). ^{1}H NMR [$(CD_3)_2SO$] δ 8.65 (s, 0.5 H), 8.64 (s, 0.5 H), 7.72 (s, 0.5 H), 7.67 (s, 0.5 H), 4.84–4.79 (m, 1 H), 4.36–4.34 (m, 4 H), 3.79–3.76 (m, 4 H), 3.68–3.53 (m, 2 H), 3.44 (s, 3 H), 3.34–3.29 (m, 2 H), 3.14 (s, 6 H), 3.04 (s, 1.6 H), 2.86 (s, 1.4 H). HRMS: calcd for $C_{17}H_{27}N_3NaO_{12}S_3$ ([M+Na]$^+$) 584.0647, found 584.0649.

5-(Bis(2-bromoethyl)amino)-*N*-(2-hydroxyethyl)-*N*-methyl-4-(methylsulfonyl)-2-nitrobenzamide (2). Reaction of compound 38 (3.72 g, 6.62 mmol) with LiBr (5.73 g, 65.9 mmol) in acetone (200 mL) , using

the same procedure as described for 1 (Method 2), provided the title compound 2 as a mixture of atropisomers and a yellow solid (3.13 g, 89%). ^{1}H NMR identical to that described previously.

((5-((2-Hydroxyethyl)(propyl)carbamoyl)-2-(methylsulfonyl)-4-nitrophenyl)azanediyl)bis(ethane-2,1-diyl) dimethanesulfonate (39). Reaction of the freshly prepared acid chloride of compound 34 (4.17 g, 7.97 mmol) with 2-(propylamino)ethanol (1.82 mL, 15.9 mmol) in DCM/THF (2:1 ratio, 105 mL), using the same procedure as described for 37, afforded the title compound 39 as a mixture of atropisomers and a yellow gum (3.81 g, 81%). ^{1}H NMR [$(CD_3)_2SO$] δ 8.66 (s, 0.4 H), 8.63 (s, 0.6 H), 7.70 (s, 0.6 H), 7.68 (s, 0.4 H), 4.82–4.78 (m, 1 H), 4.32 (br s, 4 H), 3.77–3.65 (m, 6 H), 3.50 (br s, 2 H), 3.45–3.44 (2s, 3 H), 3.17 (br s, 2 H), 3.14–3.13 (2s, 6 H), 1.67–1.59 (m, 1 H), 1.49–1.43 (m, 1 H), 0.92 (t, *J* = 7.4 Hz, 2 H) 0.68 (t, *J* = 7.3 Hz, 1 H). HRMS: calcd for $C_{19}H_{32}N_3O_{12}S_3$ ([M+H]$^+$) 590.1133, found 590.1143.

5-(Bis(2-bromoethyl)amino)-*N*-(2-hydroxyethyl)-*N*-propyl-4-(methylsulfonyl)-2-nitrobenzamide (4). Reaction of compound 39 (3.79 g, 6.43 mmol) with LiBr (11.2 g, 128.85 mmol) in acetone (150 mL), using the same procedure as described for 1 (Method 2), provided 4 as a mixture of atropisomers and a yellow solid (3.31 g, 92%). ^{1}H NMR identical to that described previously.

((5-((2-Hydroxyethyl)(ethyl)carbamoyl)-2-(ethylsulfonyl)-4-nitrophenyl)azanediyl)bis(ethane-2,1-diyl) dimethanesulfonate (42). Reaction of the freshly prepared acid chloride of compound 35 (311 mg, 0.58 mmol)with 2-(ethylamino)ethanol (254 μL, 2.61 mmol) in DCM/THF (2:1 ratio, 12 mL)using the same procedure as described for 37, afforded the title compound 42 as a mixture of atropisomers and a yellow gum (270 mg, 79%). ^{1}H NMR [$(CD_3)_2SO$] δ 8.64 (s, 0.4 H), 8.61 (s, 0.6 H), 7.69 (s, 0.6 H), 7.68 (s, 0.4 H), 4.80 (t, *J* = 5.3 Hz, 1 H), 4.35–4.33 (m, 4 H), 3.78–3.72 (m, 4 H), 3.67–3.62 (m, 2 H), 3.50 (br s, 2 H), 3.15 (s, 6 H), 2.44–2.40 (m, 2 H), 2.18–2.10 (m, 2 H), 1.18 (t, *J* = 7.0 Hz, 2 H), 1.10 (t, *J* = 7.4 Hz, 3 H), 1.02 (t, *J* = 7.0 Hz, 1 H). HRMS: calcd for $C_{19}H_{31}N_3NaO_{12}S_3$ ([M+Na]$^+$) 612.0955, found 612.0962.

5-(Bis(2-bromoethyl)amino)-4-(ethylsulfonyl)-*N*-(2-hydroxyethyl)-*N*-ethyl-2-nitrobenzamide (7). Reaction of compound 42 (270 mg, 0.46 mmol) with LiBr (797 mg, 9.16 mmol) , using the same procedure as described for 1 (Method 2), provided 7 as a mixture of atropisomers and a yellow gum (188 mg,73%). ^{1}H NMR [$(CD_3)_2SO$] δ 8.64 (s, 0.4 H), 8.62 (s, 0.6 H), 7.70 (s, 0.4 H), 7.69 (s, 0.6 H), 4.80 (t, *J* = 5.5 Hz, 0.4 H), 4.76 (t, *J* = 5.3 Hz, 0.6 H), 3.81–3.76 (m, 5 H), 3.71–3.68 (m, 3 H), 3.62–3.58 (m, 4 H), 3.49 (br s, 1 H), 3.38 (br s, 1 H), 3.21–3.15 (m, 2 H), 1.18 (t, *J* = 7.0 Hz, 2 H), 1.13–1.08 (m, 3 H), 1.02 (t, *J* = 7.0 Hz, 1 H). HRMS: calcd for $C_{17}H_{25}Br_2N_3NaO_6S$ ([M+Na]$^+$) 579.9720, found 579.9723.

4-(Propylsulfonyl)-3-fluorobenzaldehyde (13). Reaction of 3,4-difluorobenzaldehyde 10 (26.0g, 183 mmol) with sodium 1-propanesulfinate (28.6 g, 220 mmol) in DMSO (250 mL), using the same procedure as described for 12, afforded the title compound 13 as a pale yellow solid (35.5 g, 84%), M.p. 72–74 °C. ^{1}H NMR [$(CD_3)_2SO$] δ 10.10 (d, *J* = 1.5 Hz, 1 H), 8.10–8.07 (m, 1 H), 8.01–7.98 (m, 2 H), 3.38–3.44 (m, 2 H), 1.67–1.57 (m, 2 H), 0.95 (t, *J* = 7.4 Hz, 3 H). $C_{10}H_{11}FO_3S$ (Calculated): C = 52.16; H = 4.82; S = 13.93; observed: C = 52.22; H = 4.84; S = 13.89.

4-(Propylsulfonyl)-3-fluorobenzoic acid (16). Compound 13 (30.8 g, 134 mmol) was oxidized with 1.78M solution of $NaClO_2$ (16.9 g, 187 mmol) in the presence of H_2O_2 (35%, 23.0 mL, 668 mmol) in acetonitrile (280 mL) and a 0.27M buffer solution of $NaH_2PO_4.4H_2O$ (4.38 g, 28.1 mmol) and conc. HCl (2.50 mL), using the same procedure as described for 15, provided the title compound 16 as a white solid (23.8 g, 72%), M.p. 170–172 °C. ^{1}H NMR [$(CD_3)_2SO$] δ 13.84 (br s, 1 H), 8.01–7.98 (m, 2 H), 7.96–7.91 (m, 1 H), 3.47–3.41 (m, 2 H), 1.66–1.57 (m, 2 H), 0.94 (t, *J* = 7.4 Hz, 3 H). HRMS calcd for $C_{10}H_{10}FO_4S$ ([M-H]$^-$) 245.0362, found 245.0288.

4-(Propylsulfonyl)-5-fluoro-2-nitrobenzoic acid (19). Nitration of compound 16 (23.7 g, 96.2 mmol) with fuming HNO_3 (33.0 mL) and H_2SO_4 (169 mL), using the same procedure as described for 18, provided

the title compound 19 as a pale yellow solid (27.1 g, 97%), M.p. 142–145 °C. ^{1}H NMR [$(CD_3)_2SO$] δ 14.35 (br s, 1 H), 8.41 (d, *J* = 5.8 Hz, 1 H), 8.07 (d, *J* = 9.3 Hz, 1 H), 3.52–3.49 (m, 2 H), 1.72–1.62 (m, 2 H), 0.97 (t, *J* = 7.4 Hz, 3 H). HRMS: calcd for $C_{10}H_{11}FNO_6S$ ([M+H]$^+$)292.0288, found 292.0286.

tert-Butyl 4-(propylsulfonyl)-5-fluoro-2-nitrobenzoate (27). Reaction of compound 19 (25.0 g, 85.8 mmol) with *tert*-butyl acetate (200 mL) and perchloric acid (70%, 7.23 mL, 120 mmol), using the same procedure as described for 25, afforded the title compound 27 as pale yellow crystals (14.8 g, 65%), M.p. 118–119 °C. ^{1}H NMR ($CDCl_3$) δ 8.54 (d, *J* = 5.6 Hz, 1 H), 7.53 (d, *J* = 8.5 Hz, 1 H), 3.33–3.29 (m, 2 H), 1.84–1.78 (m, 2 H), 1.59 (s, 9 H), 1.07 (t, *J* = 7.4 Hz, 3 H). $C_{14}H_{18}FNO_6S$ (Calculated): C = 48.41; H = 5.22; N = 4.03; observed: C = 48.65; H = 5.22; N = 4.21. Note: The filtrate was diluted with water and treated with aqueous HCl (4M) to precipitate unreacted starting material, which was collected by filtration.

tert-Butyl 5-(bis(2-hydroxyethyl)amino)-4-(propylsulfonyl)-2-nitrobenzoate (30). Reaction of compound 27 (10.3 g, 29.7 mmol) with diethanolamine (4.01 mL, 41.6 mmol) in DMSO (22 mL), using the same procedure as described for 28, afforded the title compound 30 as a yellow gum (9.72 g, 76%). ^{1}H NMR [$(CD_3)_2SO$] δ 8.48 (s, 1 H), 7.62 (s, 1 H), 4.66 (t, *J* = 4.8 Hz, 2 H), 3.70–3.66 (m, 2 H), 3.56–3.50 (m, 8 H), 1.53 (s, 9 H), 1.48–1.42 (m, 2 H), 0.90 (t, *J* = 7.8 Hz, 3 H). HRMS: calcd for $C_{18}H_{28}N_2NaO_8S$ ([M+Na]$^+$) 455.1463, found 455.1459.

tert-Butyl 5-(bis(2-((methylsulfonyl)oxy)ethyl)amino)-4-(propylsulfonyl)-2-nitrobenzoate (33). Reaction of compound 30 (9.72 g, 22.5 mmol) with MsCl (5.20 mL, 67.4 mmol) and Et_3N (11.0 mL, 78.6 mmol), using the same procedure as described for 31, provided the title compound 33 as a yellow gum (8.82 g, 67%). ^{1}H NMR [$(CD_3)_2SO$] δ 8.50 (s, 1 H), 7.81 (s, 1 H), 4.37 (t, *J* = 5.1 Hz, 4 H), 3.77 (t, *J* = 5.0 Hz, 4 H), 3.58–3.56 (m, 2 H), 3.15 (s, 6 H), 1.60–1.54 (m, 2 H), 1.53 (s, 9 H), 0.94 (t, *J* = 7.4 Hz, 3 H). $C_{20}H_{32}N_2O_{12}S_3$ (Calculated): C = 40.81; H = 5.48; N = 4.76; observed: C = 41.01; H = 5.52; N = 4.81.

5-(Bis(2-((methylsulfonyl)oxy)ethyl)amino)-4-(propylsulfonyl)-2-nitrobenzoic acid (36). Reaction of compound 33 (8.82 g, 15.0 mmol) with TFA (25 mL) in DCM (50 mL), using the same procedure as described for 34, afforded the title compound 36 as a yellow gum (7.58 g, 95%). ^{1}H NMR [$(CD_3)_2SO$] δ 8.49 (s, 1 H), 7.87 (s, 1 H), 4.35 (t, *J* = 5.0 Hz, 4 H), 3.75 (t, *J* = 5.0 Hz, 4 H), 3.60–3.56 (m, 2 H), 3.15 (s, 6 H), 1.61–1.52 (m, 2 H), 0.94 (t, *J* = 7.4 Hz, 3 H). HRMS: calcd for $C_{16}H_{24}N_2NaO_{12}S_3$ ([M+Na]$^+$) 555.0378, found 555.0384.

((5-((2-Hydroxyethyl)carbamoyl)-2-(propylsulfonyl)-4-nitrophenyl)azanediyl)bis(ethane-2,1-diyl) dimethanesulfonate (43). Reaction of the freshly prepared acid chloride of compound 36 (518 mg, 0.94 mmol) with 2-aminoethanol (114 µL, 1.88 mmol) in DCM/THF (1:1 ratio, 16 mL), using the same procedure as described for 37, provided the title compound 43 as a yellow gum (420 mg, 78%). ^{1}H NMR [$(CD_3)_2SO$] δ 8.70 (t, *J* = 5.6 Hz, 1 H), 8.49 (s, 1 H), 7.70 (s, 1 H), 4.78 (t, *J* = 4.9 Hz, 1 H), 4.36 (t, *J* = 5.1 Hz, 4 H), 3.72 (t, *J* = 5.3 Hz, 4 H), 3.60–3.51 (m, 4 H), 3.30 (t, *J* = 6.0 Hz, 2 H), 3.17 (s, 6 H), 1.58–1.49 (m, 2 H), 0.94 (t, *J* = 7.4 Hz, 3 H). HRMS: calcd for $C_{18}H_{29}KN_3O_{12}S_3$ ([M+K]$^+$) 614.0529, found 614.0545.

5-(Bis(2-bromoethyl)amino)-4-(propylsulfonyl)-*N*-(2-hydroxyethyl)-2-nitrobenzamide (8). Reaction of compound 43 (418 mg, 0.73 mmol) with LiBr (1.27 g, 14.6 mmol), in acetone (10 ml), using the same procedure as described for 1 (Method 2), afforded 8 as a yellow solid (353 mg, 89%), M.p. 128–129 °C. ^{1}H NMR [$(CD_3)_2SO$] δ 8.76 (t, *J* = 5.6 Hz, 1 H), 8.50 (s, 1 H), 7.66 (s, 1 H), 4.79 (t, *J* = 5.4 Hz, 1 H), 3.76 (t, *J* = 7.2 Hz, 4 H), 3.69–3.62 (m, 6 H), 3.54 (q, *J* = 5.6 Hz, 2 H), 3.30 (t, *J* = 5.9 Hz, 2 H), 1.53–1.47 (m, 2 H), 0.89 (t, *J* = 7.4 Hz, 3 H). APCI MS: 546.2 ([M + H]$^+$). $C_{16}H_{23}Br_2N_3O_6S$.¹/₄ EtOAc (Calculated): C = 35.99; H = 4.44; N = 7.41; observed: C = 35.91; H = 4.32; N = 7.22.

((5-((2-Hydroxyethyl)(methyl)carbamoyl)-2-(propylsulfonyl)-4-nitrophenyl)azanediyl)bis(ethane-2,1-diyl) dimethanesulfonate (44). Reaction of the freshly prepared acid chloride of compound 36 (516 mg,

0.94 mmol) with 2-(methylamino)ethanol (150 μL, 1.88 mmol) in DCM/THF (1:1 ratio, 16 mL), using the same procedure as described for 37, provided the title compound 44 as a mixture of atropisomers and a yellow gum (352 mg, 64%). ^{1}H NMR [$(CD_3)_2SO$] δ 8.63 (s, 0.5 H), 8.62 (s, 0.5 H), 7.71 (s, 0.5 H), 7.65 (s, 0.5 H), 4.84–4.79 (m, 1 H), 4.37–4.34 (m, 4 H), 3.77 (t, *J* = 5.0 Hz, 2 H), 3.73 (br s, 2 H), 3.66 (q, *J* = 5.4 Hz, 1 H), 3.59–3.53 (m, 3 H), 3.31 (t, *J* = 6.0 Hz, 2 H), 3.15 (s, 6 H), 3.04 (s, 1.5 H), 2.85 (s, 1.5 H), 1.64–1.57 (m, 2 H), 0.96 (td, *J* = 14.9, 2.8 Hz, 3 H). HRMS: calcd for $C_{19}H_{31}KN_3O_{12}S_3$ ($[M+K]^+$) 628.0697, found 628.0701.

5-(Bis(2-bromoethyl)amino)-4-(propylsulfonyl)-*N*-(2-hydroxyethyl)-*N*-methyl-2-nitrobenzamide (9). Reaction of compound 44 (350 mg, 0.59 mmol) with LiBr (1.04 g, 12.0 mmol) in acetone (10 mL), using the same procedure as described for 1 (Method 2), afforded 9 as a mixture of atropisomers and a yellow gum (319 mg, 95%). ^{1}H NMR [$(CD_3)_2SO$] δ 8.64 (s, 0.4 H), 8.63 (s, 0.6 H), 7.69 (s, 0.6 H), 7.65 (s, 0.4 H), 4.83–4.77 (m, 1 H), 3.84–3.77 (m, 4 H), 3.66–3.54 (m, 8 H), 3.17–3.10 (br s, 2 H), 3.04 (s, 1.8 H), 2.86 (s, 1.2 H), 1.63–1.56 (m, 2 H), 0.94 (m, 3 H). HRMS: calcd for $C_{17}H_{25}Br_2N_3NaO_6S$ ($[M+Na]^+$) 579.9712, found 579.9723.

Synthesis of compound 6-P of Scheme 2

2-(5-(Bis(2-bromoethyl)amino)-*N*-methyl-4-(ethylsulfonyl)-2-nitrobenzamido)ethyl di-*tert*-butyl phosphate (47). Reaction of compound 6 (3.02 g, 5.39 mmol) with di-*tert*-butyl-*N*,*N*-diisopropylphosphoramidite (6.75 mL, 21.4 mmol) and 1*H*-tetrazole solution (3% in CH_3CN, 57.5 mL, 24.6 mmol) in DMF (4 mL) followed by oxidation with*m*-CPBA (70%, 7.70 g 43.7 mmol), using the same procedure as described for 46, afforded the title compound 47 as a mixture of atropisomers and a yellow gum (3.70 g, 89%). ^{1}H NMR [$(CD_3)_2SO$] δ 8.65 (s, 0.5 H), 8.64 (s, 0.5 H), 7.77 (s, 0.5 H), 7.59 (s, 0.5 H), 4.14–4.11 (m, 2 H), 3.84–3.81 (m, 5 H), 3.74–3.66 (m, 3 H), 3.63–3.60 (m, 4 H), 3.07 (s, 1.5 H), 2.89 (s, 1.5 H), 1.44 (s, 10 H), 1.40 (s, 8 H), 1.10 (t, *J* = 7.3 Hz, 3 H). HRMS: calcd for $C_{24}H_{40}Br_2N_3NaO_9PS$ ($[M+Na]^+$) 758.0469, found 758.0440.

2-(5-(Bis(2-bromoethyl)amino)-4-(ethylsulfonyl)-*N*-methyl-2-nitrobenzamido)ethyl dihydrogen phosphate (6-P). Reaction of compound 47 (3.70 g, 4.92 mmol) with TFA (17 mL) in DCM (17 mL), using the same procedure as described for 2-P, afforded 6-P as a mixture of atropisomers and a yellow gum (2.45 g, 87%). ^{1}H NMR [$(CD_3)_2SO$] δ 8.65 (s, 0.6 H), 8.64 (s, 0.4 H), 7.76 (s, 0.6 H), 7.62 (s, 0.4 H), 4.10-3.99 (m, 2 H), 3.84-3.80 (m, 4 H), 3.74-3.68 (m, 3 H), 3.63-3.57 (m, 5 H), 3.06 (s, 1.4 H), 2.89 (s, 1.6 H), 1.12-1.08 (m, 3 H). HRMS: calcd for $C_{16}H_{24}Br_2N_3NaO_9PS$ ($[M+Na]^+$) 645.9228, found 645.9230.

Supplementary Figure S1. Correlation between lipophilicity ($LogD_{7.4}$) and WT:NfsA_Ec IC_{50} ratio. IC_{50} values for each cell line (HCT116 WT and HCT116 NfsA_Ec) were determined as the concentration of drug required to inhibit cell growth by 50% of untreated controls following 4 hour drug exposure, with washing and regrowth for five days. R = 0.38, R^2 = 0.14.

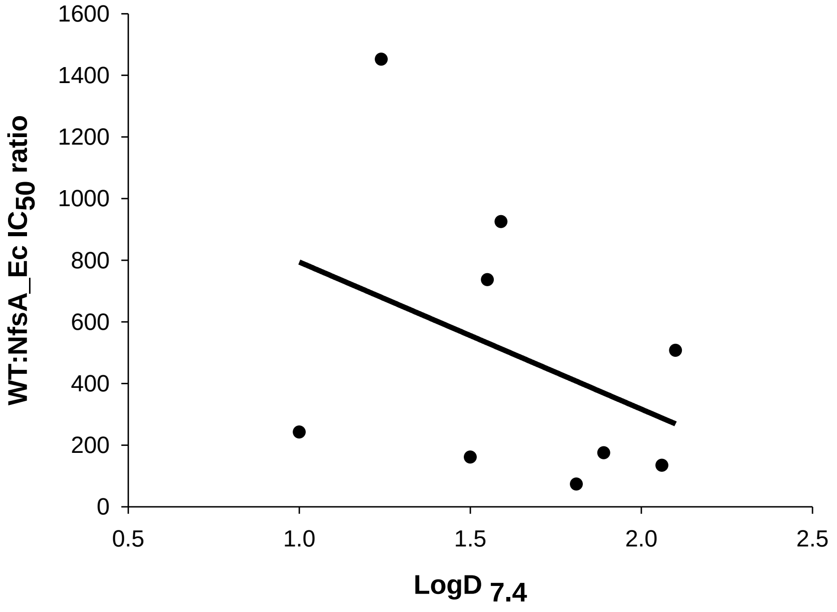

Supplementary Figure S2. Identification of alternative nitroreductases able to activate compound **6**. Bar graph displaying the fold-induction of GFP in the DNA-damage responsive *E. coli* reporter strain SOS-R4, following six-hour exposure to 20 μM of compound **6**, compared with unchallenged replicates. Individual strains expressed nitroreductase candidates derived from different bacteria, identified by a two-letter suffix as explained in the Materials and Methods. The background SOS response induced by compound **6** in an empty plasmid control strain is indicated by the dashed line. Results are the means of duplicate assays from three independent experiments, and error bars indicate ±SEM.

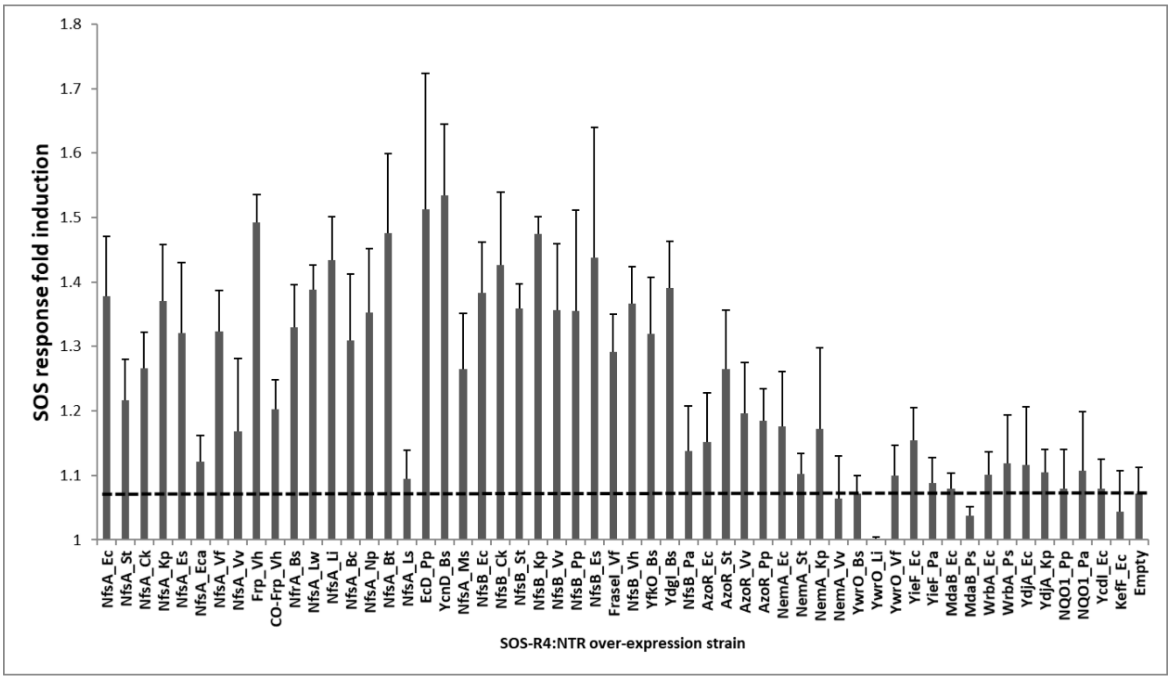

Methods:

The GFP-SOS response was determined in SOS-R4 [16] by inoculating 200 μl aliquots of LBASG (lysogeny broth supplemented with 100 μgml^{-1} ampicillin, 50 μgml^{-1} spectinomycin and 0.2% glucose (w/v)) with selected SOS-R4 nitroreductase-over-expressing strains and incubating overnight at 30 °C, 200 rpm. The next day, 20 μL of overnight culture was used to inoculate 200 μL of LBASGI (LBASG supplemented with 50 μM IPTG), which was then incubated at 30 °C, 200 rpm for 2 hours. 100 μL aliquots were transferred to 100 μL control media (LBASGI supplemented with 2% DMSO) and 100 μL challenge media (LBASGI supplemented with 40 μM (2 x final drug concentration) of compound **6** and a final concentration of 2% DMSO). Control and challenge plates were incubated at 30 °C, 200 rpm for 6 h, at which point OD_{600} and GFP fluorescence (excitation 490 nm/ emission 503 nm) were measured on an Enspire™ 2300 Multilabel Reader (Perkin Elmer, Waltham, MA). The GFP-SOS response was normalized to OD_{600} and presented as fold induction, *i.e.* the fold-increase of GFP expression from each challenged strain versus an unchallenged replicate. The bacterial species and strains from which each candidate nitroreductase gene was derived were defined by a two letter suffix, as presented in Figure

S2: Bc: *Bacillus coagulans* (36D1); Bs, *Bacillus subtilis* (ATCC 6051); Bt, *Bacillus thuringiensis* (serovar konkukian, strain 97-27), Ck, *Citrobacter koseri* (ATCC 27156); Es, *Enterobacter (Chronobacter) sakazakii* (ATCC 29544); Ec, *Erwinia carotovora* (subspecies Atrosepticum SCRI1043); Ec, *Escherichia coli* (W3110); Kp, *Klebsiella pneumoniae* (ATCC 13883); Ls, *Lactobacillus sakei* (subspecies sakei 23K); Lw, *Listeria welshimeri* (serovar 6b, strain SLCC5334); Li, *Listeria innocua* (Clip11262); Ms, *Mycobacterium smegmatis* (strain MC^{2}155); Np, *Nostoc punctiforme* (PCC 73102); Pa *Pseudomonas aeruginosa* (PAO1); Pp, *Pseudomonas putida* (KT2440); Ps, *Pseudomonas syringae pv. phaseolicola* (1448a); St, *Salmonella typhi* (ATCC l9430); Vf, *Vibrio fischeri* (ATCC 7744); Vh, *Vibrio harveyi* (ATCC 33843; Frp); Vh, *Vibrio harveyi* (HY01; CO-Frp); Vv, *Vibrio vulnificus* (ATCC 27562). The creation of the nitroreductase gene library expressed in *E. coli* reporter strain SOS-R4 was described previously [16].

Supplementary Table S1. Summary of maximum tolerated dose of test compounds in NIH-III nude mice.

		Non-tumour bearing					H1299 tumour bearing				
**Compound**	**Dose (μmol/kg)**	**n**	**BWL nadir (%)**	**Deaths**	**HEP**	**MTD**	**n**	**BWL nadir (%)**	**Deaths**	**HEP**	**MTD**
**1-P**	**1330**						5	-2.2	0	0	**Yes**
	**1780**	7	-2.8	0	0	**Yes***	8	-7.6	1	1	above
	**2370**	7	-6.9	2	0	above					
**2-P**	**750**						5	-4.0	0	0	**Yes**
	**1000**	7	-2.5	0	0	**Yes**	8	-8.7	2	0	above
	**1330**	6	-10.5	1	1	above					
**6-P**	**1330**	7	-1.8	0	0	**Yes**	8	-5.3	0	0	**Yes**
	**1780**	4	-18	0	2	above					

Footnotes: BWL nadir % = body weight loss percentage (time independent nadir). HEP = humane endpoint. * = Reference [22] herein.

Supplementary Table S2. *In vitro* anti-proliferative activity of the three lead prodrug compounds in H1299 cells expressing *E. coli* NfsA. IC50 values were determined as the concentration of drug required to inhibit cell growth by 50% of untreated controls following 4 hour drug exposure, with washing and regrowth for five days.

**Compound**	**WT**	**NfsA_Ec**	**Ratio**
**PR-104A**	131 ± 9.9	0.046 ± 0.003	2848
**1**	166 ± 26	1.18 ± 0.04	141
**2**	175 ± 3.5	0.17 ± 0.03	1029
**6**	222 ± 39	0.47 ± 0.10	472

Supplementary Table S3. Steady-state kinetic parameters* for reduction of compound **6** and PR-104A by NfsA_Ec.

**Compound**	K_m (µM)	k_{cat} (s^{-1})	k_{cat}/K_m (mM^{-1} s^{-1})
**6**	210 ± 38	5.5 ± 0.3	27 ± 5
**PR-104A**#	100 ± 11	12 ± 1.4	120 ± 14

***** Apparent kinetic parameters, measured at 250 µM NADPH

As previously reported by [15]

<u>Methods:</u>

NfsA_Ec was purified as a His_6-tagged recombinant protein as previously described [15]. Kinetics assays were performed in 60 µl in UVettes (Eppendorf), using the 2 mm light path length. Reactions contained 10 mM Tris-Cl (pH 7.0), 4% DMSO, 0.25 mM NADPH and varying concentrations of PR-104A or compound **6**. Reactions were initiated by addition of 6 µl enzyme and changes in absorbance were measured for 20 s (during linearity). For calculation of K_m and k_{cat}, substrate concentrations were varied from ~0.2 x K_m to 5 x K_m. Molar extinction coefficients of 4,800 or 6,000 M^{-1} cm^{-1} were used for compound **6** and PR-104A, respectively. Non-linear regression analysis and Michaelis-Menten curve fitting was performed using Sigmaplot 10.0 (Systat Software Inc.).
